# Evaluation of novel targeted small-molecule therapeutics in desmoplastic small round cell tumor (DSRCT) cells

**DOI:** 10.1007/s00432-026-06630-6

**Published:** 2026-10-03

**Authors:** Anne Hahn, Julia Hentschel, Shifa Saleem, Sabine Becker, Thomas Liehr, Andreas Schober, Oliver H. Krämer, Jürgen Sonnemann, Christian Marx

**Affiliations:** 1 Department of Pediatric and Adolescent Medicine, Jena University Hospital, Friedrich Schiller University Jena, Jena, Germany; 2 Institute of Human Genetics, Jena University Hospital, Friedrich Schiller University, Jena, Germany; 3 Institute for Micro and Nanotechnologies (MacroNano®), Nano-Biosystem Technology, Ilmenau University of Technology, Ilmenau, Germany; 4 Institute of Toxicology, University Medical Center, Johannes Gutenberg University Mainz, Mainz, Germany; 5 Research Center Lobeda, Jena University Hospital, Friedrich Schiller University Jena, Jena, Germany; 6 Comprehensive Cancer Center Central Germany (CCCG), Campus Jena, Jena, Germany; 7 Present Address: Faculty of Health, Medicine and Behavioural Sciences, Clem Jones Centre for Ageing Dementia Research, Queensland Brain Institute, The University of Queensland, Brisbane, 4072 Australia; 8 Present Address: Institute of Human Genetics, University of Leipzig Medical Center, Leipzig, Germany; 9 Present Address: Medical Faculty, Martin Luther University Halle-Wittenberg, Halle (Saale), Germany

**Keywords:** DSRCT, EWS-WT1, HDAC, p53, Targeted therapy, Cancer cell death

## Abstract

**Purpose:**

Desmoplastic small round cell tumor (DSRCT) is an aggressive sarcoma associated with an extremely poor prognosis. This study evaluates the therapeutic potential of targeted, non-DNA-damaging small-molecule inhibitors in an in vitro DSRCT model.

**Methods:**

We characterized the genomic architecture of the JN-DSRCT-1 cell line using fluorescence in situ hybridization (FISH) and sequencing of the tumor suppressor *TP53*. In silico analysis was performed to identify shared vulnerabilities between DSRCT and Ewing sarcoma (ES). Using flow cytometry (assessing cell death, mitochondrial membrane potential loss, caspase–3 activity, and cell cycle distribution) and quantitative real-time PCR (qPCR), we systematically evaluated a small-molecule screening panel. The panel targeted chromatin acetylation (entinostat, vorinostat, tenovin-1), p53 pathway activity (RETRA, nutlin-3, RITA), receptor tyrosine kinases (imatinib), and transcription factor DNA-binding dynamics (mithramycin).

**Results:**

JN-DSRCT-1 cells exhibited complex aneuploidy, and we confirmed the presence of the pathognomonic balanced *EWSR1::WT1* translocation. Histone deacetylase inhibitors (entinostat, vorinostat) and nutlin-3 (an MDM2 antagonist) triggered robust intrinsic apoptosis and upregulated p53 target genes (p21, PUMA, and NOXA). Mithramycin demonstrated high cytotoxicity, whereas imatinib was ineffective. Pan-caspase inhibition using z-VAD-FMK rescued cells from entinostat-, vorinostat-, nutlin-3-, and mithramycin-induced death, but failed to protect against tenovin-1, RITA, or RETRA, indicating the activation of caspase-independent cell death pathways.

**Conclusion:**

DSRCT cells display high sensitivity to histone deacetylase inhibition, p53 pathway activation, and mithramycin. Among these, histone deacetylase inhibition alone or in combination with other small molecule drugs represents a particularly promising strategy for overcoming DSRCT cell survival.

**Graphical abstract:**

**Figure:**
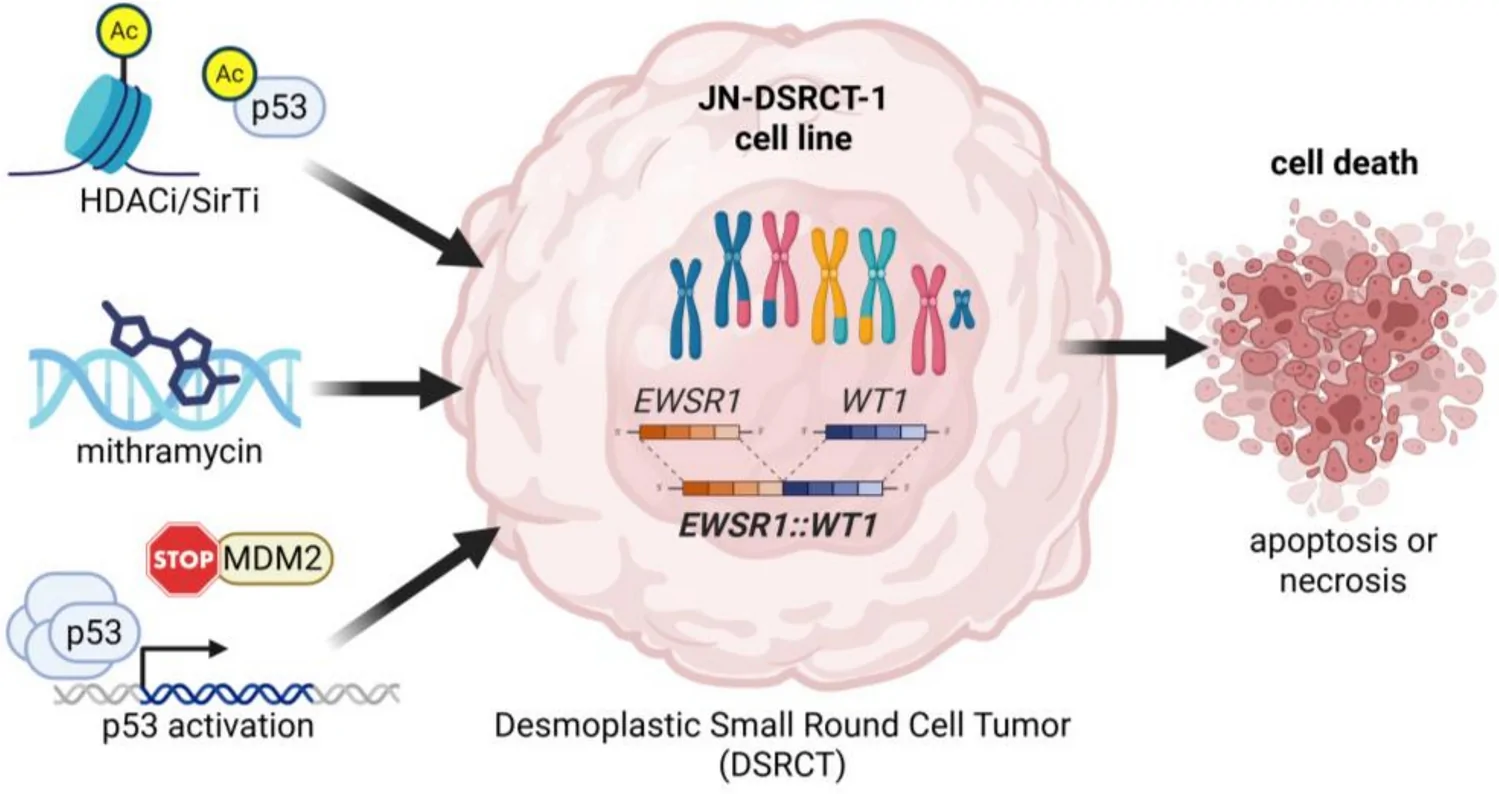
Therapeutic targeting and cell death modalities in JN-DSRCT-1 Cells. The JN-DSRCT-1 cell line serves as an in vitro model for desmoplastic small round cell tumor (DSRCT), featuring highly complex chromosomal aneuploidy alongside the pathognomonic reciprocal translocation t(11;22)(p13;q12) that generates the oncogenic EWSR1::WT1 fusion gene. The therapeutic potential of non-DNA-damaging small molecules was systematically profiled across three major molecular axes: (i) chromatin remodeling via histone deacetylase and sirtuin inhibitors (HDACi/SirTi), which promote hyperacetylation (Ac) of histones and non-histone proteins like p53; (ii) transcriptional interference via the DNA minor groove binder mithramycin; and (iii) direct p53 pathway activation through MDM2 inhibition. Exposure to these targeted compounds successfully bypasses oncogenic survival networks to trigger robust antitumor responses, ultimately driving tumor cell destruction through caspase-dependent apoptosis or caspase-independent necrosis. This figure was created using BioRender.com.

**Supplementary Information:**

The online version contains supplementary material available at https://doi.org/10.1007/s00432-026-06630-6.

## Introduction

Desmoplastic small round cell tumor (DSRCT) is a highly aggressive soft tissue sarcoma and, like Ewing sarcoma (ES), primitive neuroectodermal tumors (PNETs), and Wilms tumors (WT), is classified as a round blue cell tumor (Hovsepyan et al. 2023). The disease was first described in 1989 by Gerald and Rosai (Gerald and Rosai 1989) and is relatively rare: only about 200 cases were documented in the following 20 years (Mello et al. 2021; Miwa et al. 2010). The cellular origin of the disease is unknown; as multiple tissue markers are expressed simultaneously. Biopsies show nests of small, round, undifferentiated cells in desmoplastic stroma (Hovsepyan et al. 2023). Male patients outnumber female patients by a factor of 4–5, and the mean age of patients at the time of diagnosis is between 19 and 27 years (Gedminas et al. 2020). Symptoms are rather non-specific and include weight loss, gastrointestinal disturbances, nausea, and abdominal pain (Hovsepyan et al. 2023). Despite great effort, the disease can be brought under chronic control only in individual cases. Treatment success depends heavily on the percentage of the tumors that can be surgically removed; cytoreductive surgery is often followed by intensive multi-agent chemotherapy and postoperative whole abdominopelvic radiotherapy (Hovsepyan et al. 2023; Mello et al. 2021). Median progression-free survival ranges between 4 and 21 months, and overall survival between 17 and 60 months, with the 5-year overall survival rate in the range of 10–20% (Hovsepyan et al. 2023).

The *EWSR1* (Ewing Sarcoma Breakpoint Region 1) gene encodes the RNA-binding protein EWS, a member of the TET (TLS/EWS/TAF15) protein family (Delattre et al. 1992; Sankar and Lessnick 2011). The molecular hallmark of DSRCT is the reciprocal chromosomal translocation t(11;22) (p13; q12), typically connecting *EWSR1* in intron 7–10 on chromosome 22 and, invariably, the exons 7 and 8 of *WT1* (Wilms Tumor-1) on chromosome 11, creating the *EWSR1::WT1* fusion gene (Gedminas et al. 2020; Hovsepyan et al. 2023; Mello et al. 2021). Thereby, this translocation results in a chimeric transcription factor EWS-WT1 that regulates genes involved in cell cycle progression, exocytosis, vascularization and tissue invasion, such as the platelet-derived growth factor receptor alpha *(PDGFRA)*, and the vascular endothelial growth factor (*VEGF*) (Gedminas et al. 2020; Mello et al. 2021).

Dysregulation of histone deacetylases (HDACs) is a common feature in many cancers (Liang et al. 2023; Thomas et al. 2011). HDACs are phylogenetically divided into five classes: Classical HDACs are zinc-dependent metalloenzymes divided into classes I, II, and IV based on structural homology, expression patterns, and cellular localization (Seto and Yoshida 2014). Class I HDACs are ubiquitously expressed, nuclear-localized enzymes that deacetylate core histone proteins and repress transcription. Class II (subdivided into IIa and IIb) and class IV (only HDAC11) enzymes exhibit tissue-specific expression, dynamically shuttle or reside in the cytoplasm, and frequently target non-histone substrates (Seto and Yoshida 2014; Thomas et al. 2011). In contrast, sirtuins (SirT1–7) belonging to HDAC class III, are structurally distinct enzymes that require nicotinamide adenine dinucleotide (NAD^+^) as a cofactor, directly linking their epigenetic and metabolic activities to the nutritional state of the cell (Wu et al. 2022). HDAC and SirT inhibitors (HDACi/SirTi) can shift gene expression toward differentiation or apoptosis by increasing the acetylation both of histones and non-histone proteins, including p53 (Koppenhafer et al. 2025; Liang et al. 2023; Marx et al. 2018; Sonnemann et al. 2014), and are valuable therapeutic options beyond traditional chemotherapeutic regimens. Within a clinical study cohort evaluating *EWSR1*-rearranged round cell sarcomas (Dermawan et al. 2024), comprising 173 ES (harboring the *EWSR1::FLI1* or *EWSR1::ERG* fusion-oncogene) and 104 DSRCT cases (harboring the *EWSR1::WT1* fusion-oncogene), *ARID1A* mutations were noted in DSRCT but not in ES (in silico analysis of this cohort is included in the following sections of this study). The ARID1A protein interacts with the SWI/SNF protein complex to control chromatin structure, promotor accessibility and gene expressions; its mutations cause chromosomal alterations and cancer predisposition (Fontana et al. 2023; Fukumoto et al. 2018b). We and others have shown the effectiveness of chromatin-remodeling HDACi/SirTi in various cancer entities including ES (Koppenhafer et al. 2025; Marx et al. 2018; Sonnemann et al. 2014), which thus might also be useful for DSRCT.

Another exploitable weakness of DSRCT as of ES might be p53 upstream and downstream pathways, since *TP53* is found mutated in both tumors (Dermawan et al. 2024). We previously showed that activating p53 pathways offers promising therapeutic options for ES (Sonnemann et al. 2015, 2011), that might translate to the treatment of DSRCT cells. Inhibition of the EWS-WT1 fusion or its downstream targets is a major focus in DSRCT research (Boulay et al. 2024; Kawai-Kawachi et al. 2025). The tyrosine kinase inhibitor (TKI) imatinib is known to block BCR-ABL, c-Kit, and PDGFRA (Corless et al. 2005; Mello et al. 2021) drivers of cell cycle progression. Likewise, mithramycin, which is a DNA minor groove binding antibiotic, is known to impair EWS-FLI-mediated transcription in ES (Kaufman et al. 2026; Portugal 2024); both may influence DSRCT viability.

The objective of this study is to evaluate the effects of the HDACi entinostat, vorinostat, valproic acid, the SirTi tenovin-1, the p53 activity modulators RETRA, RITA, and nutlin-3, and of mithramycin on JN-DSRCT-1 cells. We utilized flow cytometry to measure propidium iodide (PI) uptake, the loss of mitochondrial transmembrane potential (ΔΨ_m_) and caspase−3 activity as markers of mitochondrial damage and apoptosis. Additionally, p53 target gene mRNA expression and cell cycle analysis are employed to elucidate the underlying molecular mechanisms of these compounds in DSRCT.

## Materials and methods

### Cell culture

The JN-DSRCT-1 cell line was established in 2002 by Nishio et al. (2002) and was originally derived from a tumor of a 7-year-old male patient. The fusion site of the two genes connects the *EWSR1* exon 10 and exon 8 of *WT1* (Nishio et al. 2002). JN-DSRCT-1 cells were a gift of Dr. Sean-Bong Lee (Tulane Cancer Center, LA, USA) and were maintained in conditioned medium: 1:1 mixture of DMEM and Ham's F12 medium (Lonza Group, Basel, Switzerland) supplemented with 10% fetal calf serum, 100 units/ml penicillin G sodium and 100 μg/ml streptomycin sulfate (Gibco/Invitrogen, Karlsruhe, Germany). They were cultivated at 37 °C in a humidified 5% CO_2_ incubator and routinely passaged when 90% confluent.

### Treatment of cells

Cells were seeded 24 h prior to drug exposure in tissue culture plates and treated for 24 h (qPCR) and 48 h (all other assays) with imatinib (PKC Pharmaceuticals, Woburn, MA, USA), mithramycin (PanReac/AppliChem, Darmstadt, Germany), vorinostat, entinostat, nutlin-3 (Alexis Biochemicals/Enzo Life Sciences, Lörrach, Germany), valproic acid (Sigma-Aldrich/Merck, Taufkirchen, Germany), RETRA (Tocris Bioscience, Bristol, UK), tenovin-1 or RITA (Cayman Chemical, Ann Arbor, MI, USA). If cells were treated additionally with the pan-caspase inhibitor z-VAD-FMK (zVAD; Alexis Biochemicals), the latter was added 1 h prior to treatment with other drugs.

### Flow cytometric analysis of cell death and the loss of mitochondrial transmembrane potential (ΔΨ_m_)

Analyses were done as previously described in (Marx-Blümel et al. 2017).

### Caspase−3 activity

Analyses were done as previously described in (Marx-Blümel et al. 2017).

### Cell cycle analysis

Analyses were done as previously described in (Gschwind et al. 2022).

### PCR Analysis of the EWS-FLI1 fusion gene

Analysis of EWS-WT1 expression were done as previously described in (Nishio et al. 2002).

### Fluorescence in situ hybridization (FISH)

FISH was done on chromosome preparations of the cell line JN-DSRCT-1. Chromosomes were prepared according to standard procedures (Weise and Liehr 2026). 20 metaphases, and where appropriate, also 20 interphase cells were evaluated per probe set applied. The presence of an *EWSR1* and a *WT1* gene rearrangement was checked using the commercially available probe sets ZytoLight SPEC EWSR1 Dual Color Break Apart Probe and ZytoLight SPEC WT1 Dual Color Break Apart Probe (Zytovision, Bremerhaven, Germany). A gene fusion *EWSR1* and FLI1 was confirmed using ZytoLight SPEC EWSR1/FLI1 Tricheck Probe (Zytovision, Bremerhaven, Germany). FISH was done as previously reported (Liehr 2026a).

Multicolor-FISH (M-FISH) analysis using a homemade whole chromosome painting probes (Liehr 2026b) was applied to resolve the complex karyotype of the cell line. As previously reported karyotype was determined by combining M-FISH data with inverted DAPI (4′,6-diamidino-2-phenylindole) banding.

### TP53 sequencing

Sequencing was done using as described previously (Hentschel et al. 2011). Analysis of the *TP53* gene in JN-DSRCT-1 cells was done using JSI medical systems GmbH (Kippenheim, Germany).

### Quantitative real-time PCR (qPCR)

Total RNA was isolated using Peqgold Total RNA Kit including DNase digestion (Peqlab, Erlangen, Germany). RNA was transcribed into cDNA using Omniscript (Qiagen, Hilden, Germany). Quantitative PCR was performed using the Applied Biosystems 7900HT real-time PCR system. Expression levels of p21 (*CDKN1A*) were normalized to 18S rRNA. Reactions were done in duplicate using Applied Biosystems (Darmstadt, Germany) Gene Expression Assays (18 s rRNA: Hs99999901_s1, *CDKN1A*: Hs00355782_m1, *PMAIP1*: Hs00560402_m1, *BBC3*: Hs00248075_m1) and Universal PCR Master Mix. All procedures were performed according to the manufacturer's protocols. The relative gene expressions were calculated by the 2^(ΔΔCt)^ method.

### In silico genomic analysis using cBioPortal

To explore the genomic and molecular differences between DSRCT and ES, in silico analysis was performed using the cBioPortal for Cancer Genomics framework (http://www.cbioportal.org). Genomic data were obtained from a publicly available Memorial Sloan Kettering Cancer Center cohort evaluating EWSR1-rearranged round cell sarcomas (Dermawan et al. 2024), which comprises 173 ES and 104 DSRCT cases. Clinical features, copy number alterations (CNA), mutations and structural variants of genes were compared between the two specific tumor entities using cBioPortal’s integrated exploration tools.

### Data analysis

Statistical significance of differences between experimental groups was calculated using a two-tailed Student's t-test using Microsoft 365 Excel; * = *p* < 0.05, ** = *p* < 0.01, *** = *p* < 0.001.

## Results

### Molecular cytogenetic characterization of the JN-DSRCT-1 cell line

Since its establishment in 2002 by Nishio et al. (2002), the JN-DSRCT-1 cell line has been used in research frequently, but the chromosomal complexity of these cells is still not fully understood. The *EWSR1::WT1* gene fusion serves as an exclusive diagnostic hallmark for the vast majority of DSRCT. We confirmed the fusion between *EWSR1* and *WT1* in the JN-DSRCT-1 cell line by PCR (Suppl. Fig. S1A). LNCaP cells, which were used as control, did not show a *EWSW1::WT1* PCR product, as expected. We further characterized the chromosomal complexity in JN-DSRCT-1 cells using cytogenetics and fluorescence in situ hybridization (FISH) (Suppl. Fig. S1B, C, S2 and S3). Inverted DAPI-banding and molecular cytogenetic analysis confirmed the presence of the diagnostic reciprocal translocation t(11;22)(p13;q12). This rearrangement results in the characteristic *EWSR1::WT1* gene fusion. Due to clonal evolution and numerical duplication, this locus is represented by a single derivative chromosome 11 (der(11)t(11;22)) alongside a duplicated derivative chromosome 22 (+ der(22)t(11;22)(p13;q12)), effectively yielding two copies of the der(22) fusion marker (Suppl. Fig. S1B, C and S2). Of note, we did not detect the *EWSR1::FLI1* fusion gene in JN-DSRCT-1 cells by FISH (Suppl. Fig S3).

Karyotypic analysis of the JN-DSRCT-1 cell line demonstrates a highly complex chromosomal architecture characterized by pronounced aneuploidy (chromosome count ranging from 45 to 49) and presence of multiple rearrangements (Suppl. Fig. S1B, C and S2). The karyotype could be determined as 45~49, XY, +der(5)t(5;8)(p15;p21), der(8)t(1;8)(p36;p11.1), der(11)t(11;22)(p13;q12), der(11)(11pter->11q12::8p21->8p11.1::1q12->1qter), t(17;21)(p11;p11), der(22)t(11;22)(p13;q12), +der(22)t(11;22)(p13;q12)[cp20]. Overall, these rearrangements lead to partial trisomy for chromosomes 1 and 5, 11 and 22 paired with an 8q terminal fragment, der(8), and monosomy 11 (− 11). Additionally, there is a balanced rearrangement between chromosomes 17 and 21 (der(17)t(17;21) and der(21)t(17;21)) not involving the 17p13 locus according to the FISH data, the site of *TP53*, which encodes the tumor suppressor p53 (van Kampen et al. 2025). The *TP53* status of the JN-DSRCT-1 cell line was further characterized by sequencing revealing two single nucleotide variants in this gene (Suppl. Fig. S4). The proline 72 to arginine substitution (c.215C>G, p.(Pro72Arg)) of p53 is already known to be non-pathogenic (De Souza et al. 2021). The second variant results in a heterozygous substitution of leucine 43 with phenylalanine (c.129G>C, p.(Leu43Phe)). This variant has an allele frequency of 0.00018% in the general population referring to the gnomAD database (v4.1.1; https://gnomad.broadinstitute.org/). Compared to wild-type p53 (wt-p53), p53 Leu43Phe mutants showed comparable expression levels of p53 target genes and of p21 protein following radiation in a recent publication (Abreu et al. 2024), indicating that the p53 Leu43Phe variant is functional. Therefore, this variant can be classified as likely benign according to the ClinGen VCEP (Variant Curation Expert Panel) gene-specific recommendations (https://clinicalgenome.org/affiliation/50013). Consequently, the JN-DSRCT-1 cell line serves as a validated, genetically authentic model of DSRCT, including chromosomal aberrations, and offers a suitable platform for investigating novel targeted therapeutics.

### In silico analysis of druggable targets

**Fig. 1:**
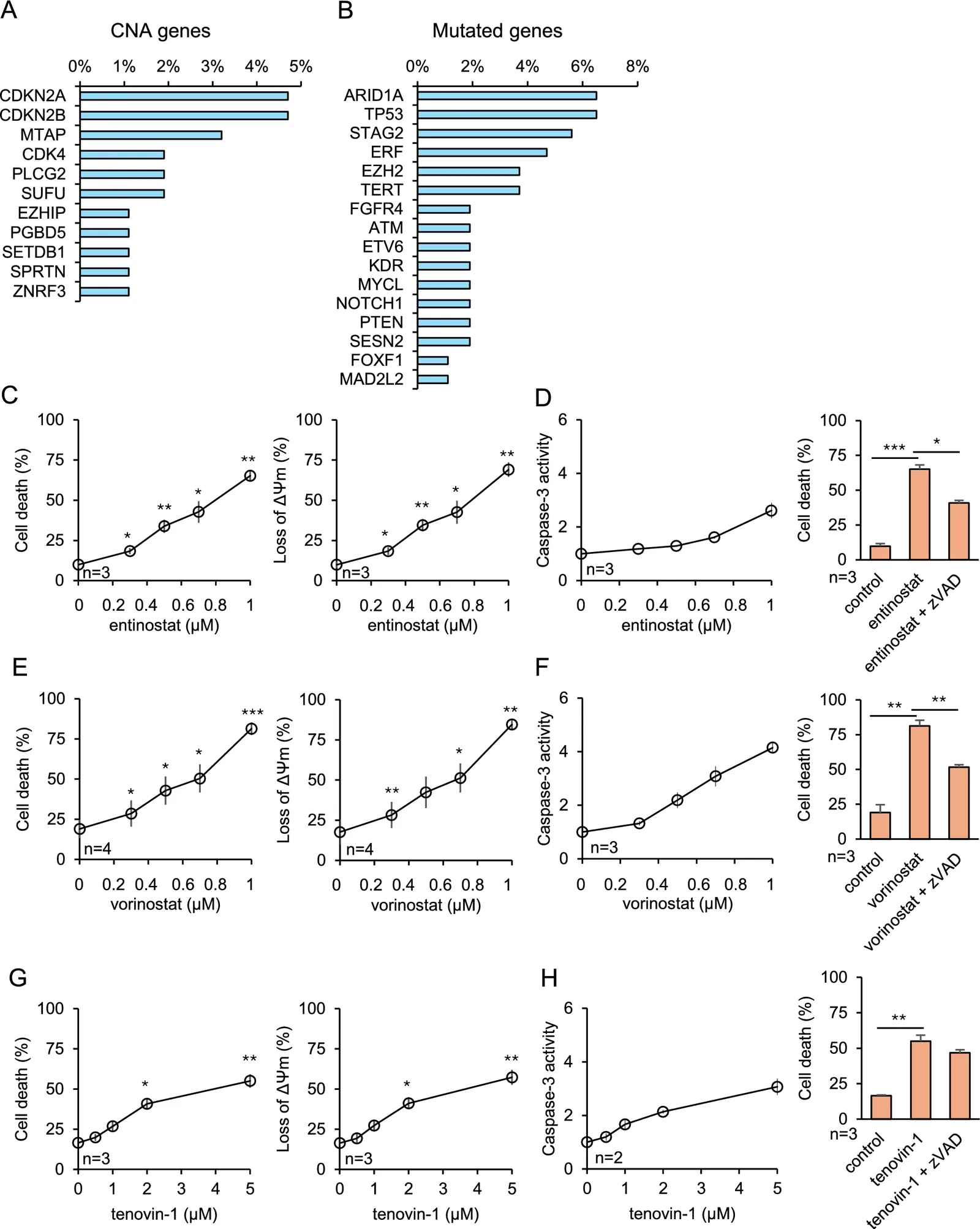
In silico characterization and cytotoxic response of JN-DSRCT-1 cells to HDACi. **A**, **B** In silico analysis was performed using the cBioPortal for Cancer Genomics framework based on the genomic data from a published Memorial Sloan Kettering Cancer Center clinical study cohort evaluating *EWSR1*-rearranged round cell sarcomas. Data for DSRCT patients is presented here as: **A** frequency of copy number alteration (CNA) variant genes; **B** frequency of mutated genes, highlighting somatic mutations. **C**–**H** Flow cytometric analyses of concentration-dependent cell death profiles, loss of mitochondrial membrane potential (ΔΨ_m_), and caspase−3 enzymatic activity assays following 48 h treatment with: 0.3–1 µM entinostat (**C**, **D**), 0.3–1 µM vorinostat (**E**, **F**) and 0.5–5 µM tenovin-1 (**G**, **H**). Right-hand bar graphs illustrate the rescue effect on cell death when the highest applied concentration was combined with 20 µM of the pan-caspase inhibitor zVAD. Data are represented as mean ±SEM; the number of replicates is included in the figure. Statistical significance is designated as: * *p* < 0.05, ** *p* < 0.01, *** *p* < 0.001

To explore druggable vulnerabilities in DSRCT, we performed an in silico analysis using the cBioPortal framework based on the genomic data from a published clinical study cohort evaluating *EWSR1*-rearranged round cell sarcomas (Dermawan et al. 2024), comprising 173 ES and 104 DSRCT cases. In silico analysis of this cohort revealed that in both ES and DSRCT the genes most frequently affected by copy number alterations (CNA) were *cyclin dependent kinase inhibitor 2A* (*CDKN2A*) and *2B* (*CDKN2B)* (Fig. 1A and S5A). *CDKN2A,* encoding p14^ARF^ and p16^INK4A^, respectively, and *CDKN2B,* encoding p15^INK4B^, regulate cell cycle progression via interaction with cyclin dependent kinases (CDKs) (Danishevich et al. 2023; Xia et al. 2021). The genomic architecture of JN-DSRCT-1 cells, however, showed a different set of CNAs (see above) and the *CDKN2A/B* locus (9p21 (Campa et al. 2016)) was copy-number neutral and structurally intact. Although rare, *TP53* and *STAG2* mutations, together with *CDKN2A* alterations are well described in ES patients and connected to a poor prognosis (Crompton et al. 2014; Tirode et al. 2014). Mirroring this, *TP53* and *STAG2* were among the mutated genes in both DSRCT and ES (Fig. 1B and S5B). The *TP53* sequencing of JN-DSRCT-1 cells confirms this (Suppl. Fig. S4). Thus, modulating p53 pathway activity might be a promising therapeutic approach for DSRCTs.

Interestingly, DSRCT patients have, among other genes, the highest mutation frequency in *ARID1A*, which is not common in ES (Fig. 1B and S5B). ARID1A belongs to the chromatin regulatory proteins and is mutated in about 10% of all human tumors of various origin (Fontana et al. 2023). In the JN-DSRCT-1 cell line, the *ARID1A* locus (1p36.11) is subject to a copy number gain, effectively present in three copies due to the partial trisomy of chromosome 1, and spatial relocation of chromosome 1, with one of those copies sitting on a structurally abnormal chromosome 8 (der(8)(1pter->1p11::8p12->8q21.3::8p22->8pter). Because the breakpoint for this translocation occurred at 1p11, the coding sequence of *ARID1A* itself is most likely intact. *ARID1A* mutations are connected to chromosomal alterations (Fontana et al. 2023; Fukumoto et al. 2018b) and might be a weakness of DSRC tumors.

Taken together, in silico analysis highlighted many overlapping gene mutations/alterations between ES and DSRCT patients, indicating that effective anti-ES therapies might also show promise in the treatment of DSRCT patients. We therefore aimed to evaluate the effects of a set of HDACi and SirTi, and of p53 pathway activity modulators, side by side with imatinib and mithramycin in a small molecule drug screening approach on JN-DSRCT-1 cells.

### Experimental evaluation of entinostat in JN-DSRCT-1 cells

To determine the effectiveness of the class I/class IV HDACi entinostat (MS-275) (Thomas et al. 2011), PI uptake as marker for cell death was measured by flow cytometry. Unless specified otherwise, JN-DSRCT-1 cells were exposed to all small molecule agents for 48 h prior to analysis throughout. A narrow concentration gradient was trialed, causing a clear and significant concentration-dependent increase in cell death (65.2% at 1 µM) (Fig. 1C). The loss of ΔΨ_m_ is a crucial event in intrinsic apoptosis execution and typically precedes large-scale genomic DNA fragmentation (Zamzami et al. 1995); it triggers the release of cytochrome c into the cytosol, initiating the downstream caspase cascade (Dho et al. 2025). The loss of ΔΨ_m_ was assessed by flow cytometric using DiOC6(3) that ceases to accumulate in mitochondria of cells undergoing apoptosis. Increasing entinostat concentrations led to a significant and progressive ΔΨ_m_ loss (69.1% at 1 µM) (Fig. 1C). The enzymatic activity of caspase−3, which we measured using the fluorogenic substrate Ac-DEVD-AMC, increased up to 2.6-fold relative to untreated cells (at 1 µM) (Fig. 1D). To further characterize its mechanism of action and delineate the role of caspase-dependent cell death, entinostat was combined with the pan-caspase inhibitor zVAD, which significantly reduced cell death by 24.3% (Fig. 1D), but had negligible impact on ΔΨ_m_ loss (Suppl. Fig. S6A). This observation matches the classical model where the upstream mitochondrial outer membrane permeabilization (MOMP) occurs independently, or upstream of, effector caspase activity (Tait and Green 2008).

### Evaluation of further HDACi therapeutics

To contextualize the effectiveness of entinostat, other HDACi/SirTi were evaluated under identical experimental conditions. The class I, II and IV HDACi vorinostat (SAHA) (Thomas et al. 2011) induced a significant, concentration-dependent increase in cell death (81.4% at 1 µM) and loss of ΔΨ_m_ (84.6% at 1 µM) (Fig. 1E). Congruently, 1 μM vorinostat stimulated a 4-fold increase in caspase−3 activation; co-treatment with zVAD significantly reduced total cell death by 29.8%, with only marginal effects on the loss of ΔΨ_m_ (Fig. 1F and S6A). The spot-check analysis of the class I and II HDACi valproic acid (VPA) (Thomas et al. 2011) confirmed the effectiveness of HDACi in JN-DSRCT-1 cells (Suppl. Fig. S6B-C). VPA induced a significant concentration-dependent increase in cell death (63.7% at 2 mM) and loss of ΔΨ_m_ (67.2% at 2 mM), which was accompanied by a 4.5-fold increase in caspase−3 activation (at 2 mM).

Finally, we tested class III HDACi (SirTi) to find the most relevant HDAC class in JN-DSRCT-1 cells. Sirtuins can deacetylate p53, thereby suppressing its pro-apoptotic function. This halts transition into senescence, while control mechanisms of cell cycle progression are simultaneously disabled (Marx et al. 2018). The SirT1/2 inhibitor tenovin-1 displayed a significant concentration–response effect; however, total cytotoxicity plateaued at 5 µM (55% cell death and 57.3% ΔΨ_m_ loss at 5 µM) (Fig. 1G). Saturation kinetics of tenovin-1 beginning at 3 μM have been revealed in previous screenings in various ES cell lines (Marx et al. 2018). Upon exposure to 5 μM tenovin-1, caspase−3 activity rose 5-fold, however, co-treatment with zVAD failed to reduce cell death and ΔΨ_m_ loss (Fig. 1H and S6A), indicating that tenovin-1-mediated cytotoxicity does not rely on caspase activation in JN-DSRCT-1 cells.

### Restoring p53 pathway activity as therapeutic option

**Fig. 2:**
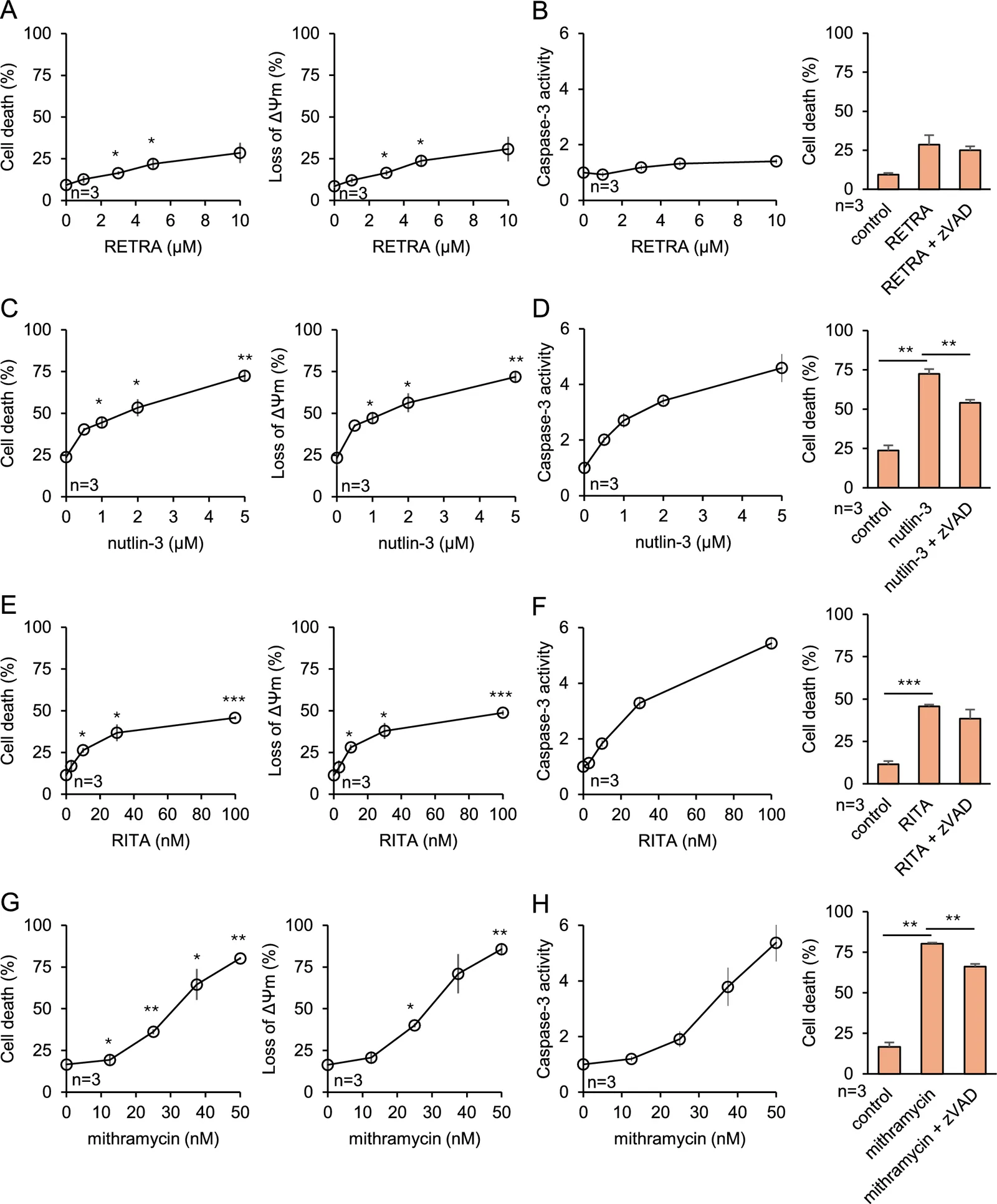
Assessment of small-molecule induced apoptosis via p53 pathway activation or mithramycin. **A**–**H** Flow cytometric analyses of concentration-dependent cell death profiles, loss of ΔΨ_m_, and caspase−3 enzymatic activity assays following 48 h treatment with: 1–10 µM RETRA (**A**, **B**), 0.5–5.0 µM nutlin-3 (**C**, **D**), 3–100 nM RITA (**E**, **F**) and 12.5–50.0 nM mithramycin (**G**, **H**). Right-hand bar graphs illustrate the rescue effect on cell death when the highest applied concentration was combined with 20 µM of the pan-caspase inhibitor zVAD. Data are represented as mean ±SEM; the number of replicates is given within the figure. Statistical significance is designated as: * *p* < 0.05, ** *p* < 0.01, *** *p* < 0.001

Tenovin-1 protects p53 from deacetylation and degradation, increasing its activity primarily in cells with wt-p53 (Lain et al. 2008; Marx et al. 2018; Sonnemann et al. 2014). (Re)activation of p53 and its downstream signaling is a key strategy for inducing apoptosis in malignant cells. This is often achieved by disrupting the inhibitory interaction of MDM2 with p53 (Wang et al. 2023). P63 and p73, members of the p53 family, have partial functional redundancy with p53. Some p53 mutants (mt-p53) lack tumor-suppressive properties, but interact with and sequester p73 and p63 (Klein et al. 2021; Wolf et al. 2018). The compound RETRA is designed to minimize the inhibitory effects of mt-p53 on p73, thereby restoring tumor-suppressive pathways in mt-p53 cells (Kravchenko et al. 2008; Sonnemann et al. 2015). The concentrations of RETRA tested in this screen displayed moderate anti-tumor effectiveness, with overall cell death of 28.5% and ΔΨ_m_ loss in 30.8% of cells, both plateauing at 10 µM (Fig. 2A). Caspase−3 activation (1.4-fold at 10 µM) was weak compared to other compounds and co-treatment with zVAD yielded no significant reduction in cell death and ΔΨ_m_ loss (Fig. 2B and S6D).

Nutlin-3 (4,5-dihydroimidazole), an MDM2 antagonist causing the stabilization and activation of p53 (Shen and Maki 2011), has a significant apoptotic influence on ES cell lines (Sonnemann et al. 2011). In our present small-molecule screen, nutlin-3 proved to be a highly potent agent, inducing up to 72.5% cell death and 71.8% ΔΨ_m_ loss in JN-DSRCT-1 cells at low micromolar concentrations (5 µM) (Fig. 2C). This correlated with a strong 4.6-fold induction of caspase−3 activity; combining nutlin-3 with zVAD reduced cell death by 18.3% and the loss of ΔΨ_m_ by 8.2% (Fig. 2D and S6D).

The molecule RITA inhibits the interaction of MDM2 and MDMX with wt-p53 or mt-p53 and only moderately affects non-malignant cells (Burmakin et al. 2013; Zhan et al. 2022). RITA demonstrated potency at nanomolar concentrations, inducing a concentration-dependent response that plateaued at 100 nM (45.8% cell death, 48.7% ΔΨ_m_ loss and a 5.4-fold caspase−3 activation) (Fig. 2E, F). Co-treatment with zVAD failed to reduce cell death and ΔΨ_m_ loss (Fig. 2F and S6D), indicating that RITA’s primary mechanism of cell death is driven by alternative, caspase-independent pathways.

### Comparative evaluation of alternative therapeutic agents

We asked if other therapeutics beyond chromatin remodeling and p53 activation would be effective in JN-DSRCT-1 cells. Mithramycin, shown to be very effective in ES (Kaufman et al. 2026; Portugal 2024), emerged as one of the most potent cytotoxic agents in our experimental panel. At just 50 nM, it triggered 80.2% cell death, ΔΨ_m_ loss in 85.7% of cells (Fig. 2G) and a 5.4-fold caspase−3 activation (Fig. 2H). Co-treatment with zVAD reduced total cell death by 14% (Fig. 2H), while providing only mild reduction of ΔΨ_m_ loss (Suppl. Fig. S6F). In contrast, the TKI imatinib demonstrated no meaningful therapeutic effectiveness against JN-DSRCT-1 cells. Across all evaluated concentrations, the fraction of dead cells and of cells exhibiting a loss of ΔΨ_m_ remained at a baseline level of 10–15% (Suppl. Fig. S6E). We therefore discontinued the use of imatinib in this screen.

### Analyses of cell cycle profiles and gene expression after HDACi treatment

**Fig. 3:**
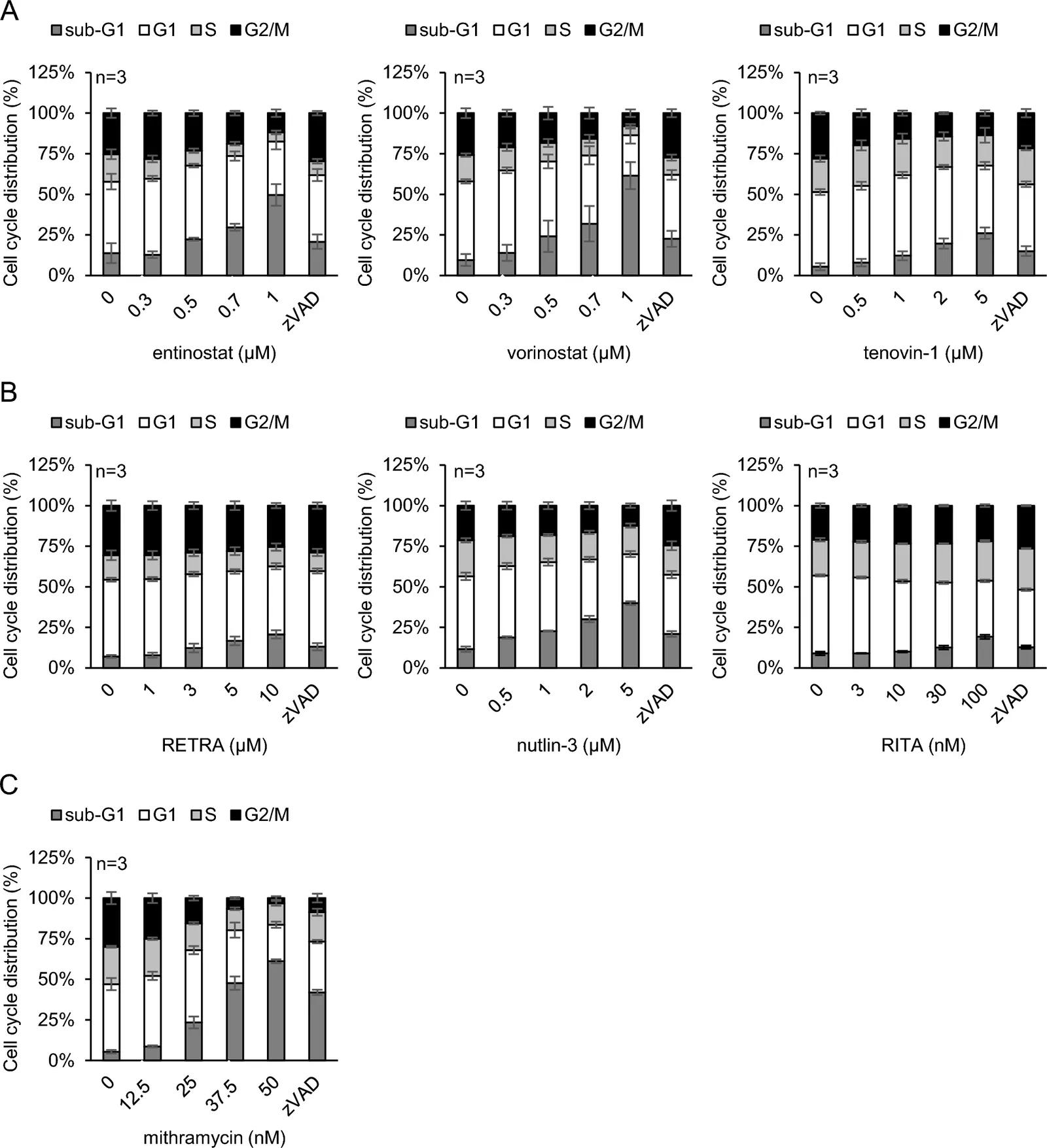
Cell cycle dynamics and redistribution triggered by target compounds. **A**–**C** Flow cytometric analyses of cell cycle distribution quantifying the percentage of cells in sub-G1, G1, S, and G2/M phases. Cells were analyzed after 48 h exposure to increasing concentrations of: **A** HDACi/SirTi: entinostat, vorinostat and tenovin-1; **B** p53 pathway activators: RETRA, nutlin-3 and RITA; and **C** mithramycin; respective concentrations are depicted in the graph. The rescue effect on cell cycle distribution when the highest applied concentration was combined with 20 µM of zVAD is depicted within the graphs. Accumulation in the sub-G1 fraction marks apoptotic DNA fragmentation. Data are represented as mean ±SEM; the number of replicates is included in the figure

P53 is a primary upstream regulator of caspase activation, DNA repair, and cell cycle arrest (Dho et al. 2025; Sullivan et al. 2018; Wang et al. 2023). Thus, we speculated that JN-DSRCT-1 cells might undergo cell cycle perturbations following treatments within this small-molecule screening panel. Cells were analyzed via flow cytometry-based quantitative DNA content profiling, which identified four distinct populations: sub-G1 (fragmented DNA, indicative for apoptosis), G1 (2n DNA content), S (replicating DNA), and G2/M-phase (4n DNA content, prior to cell division). In untreated conditions, most cells reside in G1-phase (Fig. 3). With increasing entinostat concentrations, accumulation of cells in the sub-G1 fraction occurred (Fig. 3A); the percentage of cells remaining in the G1-phase remained largely stable. Concurrently, S-phase and G2/M-phase populations decreased (Fig. 3A). Co-treatment with zVAD decreased the sub-G1 fraction, confirming previous experiments (Figs. 1D and 3A and S6A). To evaluate the upstream activation of p53, expression levels of its targets cyclin-dependent kinase inhibitor p21 (gene name: *CDKN1A*), p53 upregulated modulator of apoptosis (PUMA; gene name: *BBC3*) and NOXA (gene name: *PMAIP1*) (Sullivan et al. 2018; Wang et al. 2021) were assessed by qPCR (Suppl. Fig. S6G). Total RNA was isolated after 24 h treatment with entinostat. This time point precedes the full cytotoxicity that entinostat unfolds at 48 h (Fig. 1C). Treated cells exhibited a 1.7-fold increase in *p21* and *PUMA*, and a 2.5-fold increase in *NOXA* mRNA expression (Suppl. Fig. S6G), showing a weak but notable activation of p53-dependent gene expression in JN-DSRCT-1 cells.

Following treatment with vorinostat, cell cycle analysis tracked a concentration-dependent increase in the sub-G1 fraction at the expense of other phases; however, the relative ratio between G1, S, and G2/M remained uniform, indicating the absence of a true cell cycle arrest (Fig. 3A). As seen with entinostat, co-treatment of vorinostat with zVAD reduced the sub-G1 apoptotic fraction (Fig. 3A). Furthermore, vorinostat induced a 1.5-fold upregulation of *p21* and *NOXA*, and a 1.3-fold increase in *PUMA* mRNA expression (Suppl. Fig. S6G). Cell cycle dynamics showed a concentration-dependent reduction in S and G2/M populations after tenovin-1 treatment, while the G1 fraction remained stable around 46–49% (Fig. 3A). qPCR revealed a strong 3.5-fold upregulation of *p21*, 3-fold increase of *PUMA* and a 1.4-fold increase of *NOXA* mRNA transcripts (Suppl. Fig. S6G), indicating a strong transcriptional activation of p53.

### Cell cycle profiling after p53 activation or mithramycin treatment

The p53 signaling (re)activators RETRA, nutlin-3 and RITA induced a uniform, proportional decrease across G1, S, and G2/M-phases shifting cells into the sub-G1 fraction in a concentration-dependent manner, with nutlin-3 showing strongest effects (Fig. 3B). This indicated that RETRA, nutlin-3 or RITA induced apoptosis/cell death rather than an imminent cell cycle arrest. qPCR showed a 1.4-fold increase in *p21* and *PUMA*, and 1.6-fold upregulation of *NOXA* mRNA expression following RETRA treatment and a 2.1-fold increase in *p21*, a 1.9-fold increase in *PUMA*, and 1.2-fold upregulation of *NOXA* mRNA expression following RITA treatment (Suppl. Fig. S6G). Again, nutlin-3 induced the strongest increase in p53-dependent target gene transcription among these three agents: 4-fold in *p21*, 2.7-fold in *PUMA* and 1.4-fold in *NOXA* (Suppl. Fig. S6G).

Mithramycin triggered a robust expansion of the sub-G1 population, which was accompanied by a disproportionately large decline in the G2/M phase compared to the S-phase—indicating a unique disruption of cell cycle distribution (Fig. 3C). While the G1 population remained stable at lower concentrations, it dropped sharply at higher concentrations as the number of sub-G1 cells surged (Fig. 3C). Furthermore, qPCR assays revealed that mithramycin treatment substantially upregulated apoptotic and cell-cycle transcripts, yielding a 2.4-fold increase in *p21*, a 1.9-fold increase in *PUMA*, and a 1.3-fold increase in *NOXA* (Suppl. Fig. S6G), indicating a strong transcriptional activation of p53 comparable to nutlin-3 and tenovin-1.

## Discussion

DSRCT is a rare and highly aggressive sarcoma driven by the pathogenic t(11;22)(p13;q12) translocation, which expresses the oncogenic transcription factor EWS-WT1, driving dysregulated gene expression and DSRCT development (Boulay et al. 2024; Gedminas et al. 2020; Hovsepyan et al. 2023; Mello et al. 2021). We characterized the genomic landscape of the JN-DSRCT-1 cell line in detail, performed an in silico analysis of clinical cohort data from DSRCT and ES patients to find vulnerabilities and confirmed the cell line to be a representative and valid model for DSRCT, including chromosomal complexity, key gene alterations and the *EWSR1::WT1* gene fusion. Because intensive multi-agent treatment regimens rarely secure long-term overall patient survival, identifying non-DNA-damaging small-molecule therapies is clinically paramount (Hovsepyan et al. 2023). This study systematically profiled the landscape of promising therapeutics to target the JN-DSRCT-1 cell line, revealing profound sensitivity to epigenetic modifiers alongside direct p53 pathway activators, and the DNA-binding agent mithramycin (Table 1).

**Table 1:** Summary of small-molecule profiling in the JN-DSRCT-1 cell line

Compound	Effective concentration	Max. cell death	Max. ΔΨ_*m*_ loss	Caspase−3 induction	zVAD effect	p21 expression
Entinostat	0.3–1.0 µM	~65%	~69%	2.6-fold	yes	1.7-fold
Vorinostat	0.3–1.0 µM	~81%	~85%	4.0-fold	yes	1.5-fold
Tenovin-1	0.5–5.0 µM	~55%	~57%	3.5-fold	no	3.5-fold
RETRA	1–10 µM	~28%	~31%	1.4-fold	no	1.4-fold
Nutlin-3	0.5–5.0 µM	~73%	~72%	4.6-fold	yes	4-fold
RITA	3–100 nM	~46%	~49%	5.4-fold	no	2.1-fold
Mithramycin	12.5–50.0 nM	~80%	~86%	5.4-fold	yes	2.4-fold

Our screening revealed distinct variations in how small molecules effect cell death, highlighting critical therapeutic bottlenecks and resistance mechanisms. The absolute failure of the TKI imatinib to modulate viability aligns with data from DSRCT patients enrolled in clinical trials (Bexelius et al. 2020; Chao et al. 2010). This confirms that DSRCT cell survival depends on deep-seated transcriptional and epigenetic networks rather than upstream surface-receptor tyrosine kinase signaling. In contrast, mithramycin emerged, together with class I/class II HDACi and nutlin-3, as one of the most potent cytotoxic agents in our panel, driving robust cell death at low nanomolar concentrations (Table 1). Since mithramycin binds the minor groove of DNA at GC/GG-rich sequences, it has an influence on different transcription factors, particularly in tumors with deficiencies of the chromatin remodeling SWI/SNF protein complex (Kaufman et al. 2026; Portugal 2024). Thus, mithramycin-induced chromatin alterations and changes in gene expression patterns might also contribute to in the cytotoxicity of this agent.

The elevated frequency of *ARID1A* and *TP53* mutations in DSRCT (Dermawan et al. 2024) might be a key weakness of this cancer. Among the tested HDACi, we see the high potency of class I/class II HDACs in impacting DSRCT survival, which primarily deacetylate histone proteins and thus, regulate chromatin structures and gene expression patterns (Seto and Yoshida 2014; Thomas et al. 2011). This corresponds to *ARID1A* mutations in DSRCTs and related chromosomal structure alterations, making DSRCTs vulnerable against class I/class II HDACi (Fukumoto et al. 2018a, 2018b; Megino-Luque et al. 2022). EWS-WT1 promotes the activation of the anti-apoptotic, pro-proliferative transcription factors STAT3 and STAT5 in DSCRT cells (Wong et al. 2002). Future studies are necessary to show if the impact of these HDACi on JAK-STAT signaling (Mustafa and Krämer 2023) can explain their toxic effects on such cells. Treatment with classical HDAC inhibitors (entinostat and vorinostat), as well as with nutlin-3, or mithramycin triggered intrinsic apoptosis, characterized by a loss of ΔΨ_m_, caspase−3 activation, and sub-G1 DNA fragmentation. Co-treatment with the pan-caspase inhibitor zVAD significantly reduced overall cell death (Table 1), confirming a caspase-mediated cascade downstream of ΔΨ_m_ loss (Tait and Green 2008).

Conversely, zVAD completely failed to rescue cell viability following treatment with the SirTi tenovin-1, and p53 pathway activators RITA, or RETRA (Table 1). While these compounds strongly activated caspase−3 independently, their cytotoxicity remained unhalted by caspase inhibition, indicating that alternative, programmed necrotic or necroptotic pathways become dominant when intrinsic apoptosis is blocked (Kroemer and Martin 2005; Vandenabeele et al. 2006). Notably, for apoptotic-dominant agents (entinostat, vorinostat, nutlin-3 and mithramycin), the overall percentage of dead cells consistently exceeded the sub-G1 fraction. This decoupling, combined with the fact that zVAD suppressed DNA fragmentation better than total cell death, indicates that a substantial subpopulation of DSRCT cells undergoes parallel, caspase-independent necrosis (Kroemer and Martin 2005; Vandenabeele et al. 2006). P53- and caspase-independent cell death mechanisms in various cancer entities are well documented by our group and involve, for example, apoptosis-inducing factor (AIF) signaling, endoplasmic reticulum stress and autophagy (Gschwind et al. 2022; Marx et al. 2018, 2021; Sonnemann et al. 2015, 2014). Hence, alternative cell death pathways become dominant after caspase inhibition in DSRCT. Relying solely on caspase-dependent mechanisms in the clinic may therefore leave therapies vulnerable to resistance if downstream apoptotic checkpoints mutate.

Cell cycle profiling in this screening showed that p53 pathway activators (RETRA, nutlin-3 and RITA) did not change cell cycle profiles per se, although they increased the sub-G1 fraction of cells. However, the chromatin remodeling HDACi (entinostat and vorinostat) together with the DNA-binding mithramycin induced a concentration-dependent cell cycle arrest in parallel to increased sub-G1 cell fractions. This might be a relevant aspect for designing novel therapies for DSRCT, particularly when combining these agents with classical DNA-damaging chemotherapy or cell cycle inhibitors, such as CDK inhibitors.

The status of p53 and its function in DSRCT has been historically debated; while in silico analysis and sequencing confirmed a high frequency of *TP53* mutations across DSRCT cohorts, our transcriptional profiling indicates that p53 pathways in JN-DSRCT-1 cells remain functional despite two single nucleotide variants of *TP53*. Treatment with HDAC/SirT inhibitors, MDM2 antagonists, and mithramycin uniformly triggered a statistically significant upregulation of the canonical p53 target genes (Sullivan et al. 2018; Wang et al. 2021) *CDKN1A* (p21), *BBC3* (PUMA), and *PMAIP1* (NOXA) prior to structural cell death. Interestingly, tenovin-1, which activates wt-p53 (Lain et al. 2008; Marx et al. 2018; Sonnemann et al. 2014), together with p53 activators (RITA and nutlin-3) and mithramycin induced the strongest increase in p53 target gene transcription (Table 1), potentially eliciting a positive effect on tumor-suppression. In addition, we could show that RETRA, which activates p63 and p73 pathways in cancer cells with mt-p53 (Kravchenko et al. 2008; Sonnemann et al. 2015), is ineffective in JN-DSRCT-1 cells compared to nutlin-3 and RITA (Burmakin et al. 2013; Shen and Maki 2011; Sonnemann et al. 2011; Zhan et al. 2022), which directly activate p53 signaling. This provides clear evidence that DSRC tumors can harbor intact wt-p53 pathways that can be therapeutically exploited.

While our in vitro findings demonstrate clear cytotoxic efficacy for class I/class II HDAC inhibitors, nutlin-3 and mithramycin in DSRCT cells, these responses should be validated in complex physiological environments. In particular, confirming whether single-agent potency translates into sustained tumor regression and acceptable tolerability in vivo represents a critical next step. Evaluating these small-molecule compounds in xenograft models will be essential to establish pharmacodynamic biomarkers, assess off-target toxicities, and determine effective therapeutic windows for clinical translation.

To date, several HDACi have successfully transitioned into clinical practice, including vorinostat, romidepsin, belinostat, panabinostat (no longer FDA approved), givinostat, and chidamide (Iyer et al. a^2026^; Mustafa and Krämer 2023). Although regulatory approvals vary globally across agencies such as the United States Food and Drug Administration (FDA), European Medicines Agency (EMA), National Medical Products Administration (NMPA) in China and Pharmaceuticals and Medical Devices Agency (PMDA) in Japan, these agents already demonstrate promising therapeutic potential of targeting histone deacetylation in cancer patients (Iyer et al. 2026; Mustafa and Krämer 2023) and might be applied to DSRCT patients in future. From a clinical perspective, despite its universal significance as a tumor suppressor, p53 still remains considered 'undruggable' because restoring mutant protein function presents a greater therapeutic challenge than inhibiting oncogenic activity (Wang et al. 2026). Although various p53 reactivation strategies entered clinical trials over the past decades, none obtained regulatory approval (Wang et al. 2026), reducing their therapeutic potential for DSRCT.

## Conclusion

To advance toward biomarker-driven, universally applicable clinical protocols and move away from purely experimental, compassionate-use therapies, the pre-clinical framework must evolve to integrate multi-clonal cellular models in the future. Epigenetic modifiers like entinostat or vorinostat alone or in combination with DNA-binding drugs like mithramycin, classical DNA-damaging chemotherapy or cell cycle inhibitors, such as CDK inhibitors, represents a highly promising, multi-agent strategy to bypass downstream apoptotic resistance, prevent alternative necrotic escape pathways, and effectively eliminate DSRCT.

## Limitations

The primary limitation of current pre-clinical DSRCT research is the heavy reliance on a few, long-established cell lines, including JN-DSRCT-1 cells, used as the sole model for the analysis shown in this study. DSRCT is an incredibly heterogeneous disease; individual patient tumors possess unique structural fusion splice variants, varying CNAs, and distinct stromal microenvironments (Boulay et al. 2024; Dermawan et al. 2024), which makes representative in vivo models significantly more difficult to establish. Because DSRCT is rare, clinical trials are virtually impossible to conduct in a prospective, randomized fashion, leaving the oncological community reliant on retrospective analyses (Hovsepyan et al. 2023).

## Supplementary Information

Below is the link to the electronic supplementary material.

Supplementary file1 (PDF 570 KB)
Supplementary figure S1: Molecular profiling of the EWSR1::WT1 translocation. A) Agarose gel electrophoresis for the separation of PCR products (EWSR1::WT1 fusion junction at 449 bp and β-actin). Lane 1: marker; lane 3: fusion junction in DSRCT cDNA; lane 4: fusion junction in LNCaP cDNA; lane 5: fusion junction blank control (H2O); lane 6: β-actin in DSRCT cDNA; lane 7: β-actin in LNCaP cDNA; lane 8: β-actin blank control (H2O). LNCaP cells were used as a negative control and β-actin as an internal loading reference. B) FISH micrograph using a break-apart probe system on metaphase and interphase chromosomes to confirm the structural genomic rearrangement of the EWSR1 locus (22q12.2). C) In-scale genomic physical probe map mapping hybridization coordinates of the SPEC EWSR1 dual-color probe set flanking the breakpoint region.
Supplementary figure S2: M-FISH and WT1 locus mapping. A) Molecular verification mapping the translocation t(11;22)(p13;q12) yielding the functional EWSR1::WT1 fusion locus. B) Whole-genome M-FISH and inverted DAPI-banded chromosomes of the JN-DSRCT-1 cell line showing numerical and complex structural alterations, alongside a dedicated physical probe map detailing hybridization sites across the WT1 gene region on chromosome 11p13.
Supplementary figure S3: Dissection of EWSR1::FLI1 loci. Comparative multi-channel fluorescence imaging using specific aqua, orange, and green filters illustrating a non-rearranged structural configuration versus a positive break-apart signal resulting from an alternative t(11;22)(q24;q12) EWSR1::FLI1 translocation event.
Supplementary figure S4: Sequencing chromatogram and mutational profile of the TP53 gene. High-resolution capillary sequencing trace files and a summary mutation data table confirming the existence of a heterozygous missense variant at nucleotide position 33 (c.129G>C) causing a Leu-to-Phe substitution (L43F) in the p53 protein, alongside a homozygous non-pathogenic single nucleotide polymorphism at position 119 (SNP rs1042522, c.215C>G) within Exon 4 of the TP53 gene, causing a Pro-to-Arg substitution (P72R) in the p53 protein in JN-DSRCT-1 cells.
Supplementary figure S5: Comparative profiles of copy number alterations and somatic variants in Ewing Sarcoma. A-B) In silico analysis was performed using the cBioPortal for Cancer Genomics framework based on the genomic data from a published Memorial Sloan Kettering Cancer Center clinical study cohort evaluating EWSR1-rearranged round cell sarcomas. Data for ES patients is presented here as: A) frequency of CNA variant genes; B) frequency of mutated genes, highlighting somatic mutations.
Supplementary figure S6: Profiling mitochondria integrity and transcriptional activation of downstream p53 target genes. Flow cytometric analyses of: A) ΔΨm loss following 48 h treatment with 1 µM entinostat, 1 µM vorinostat or 5 µM tenovin-1 in combination with 20 µM zVAD. B-C) concentration-dependent profiles of cell death, ΔΨm loss, and caspase-3 enzymatic activity assays following 48 h treatment with 0.2-2 mM valproic acid (VPA). D) ΔΨm loss following 48 h treatment with 10 µM RETRA, 5.0 µM nutlin-3 and 100 nM RITA in combination with 20 µM zVAD. E) concentration-dependent profiles of cell death and ΔΨm loss following 48 h treatment with 0.1-10 µM imatinib. F) ΔΨm loss following 48 h treatment with 50.0 nM mithramycin in combination with 20 µM zVAD. G) qPCR transcription profiling showing the relative fold-change expression levels of the functional p53-responsive transcripts CDKN1A (p21), BBC3 (PUMA), and PMAIP1 (NOXA) across JN-DSRCT-1 cells treated with the indicated small molecules for 24 h. Data are represented as mean ±SEM; the number of replicates is given within the figure. Statistical significance is designated as: * p < 0.05, ** p < 0.01, *** p < 0.001.

## Data Availability

The data that support the findings of this study are available from the corresponding author up-on reasonable request.

## Abbreviations

AIF: Apoptosis-inducing factor
CNA: Copy number alterations
CDK: Cyclin dependent kinase
CDKN2A: Cyclin dependent kinase inhibitor 2A
CDKN2B: Cyclin dependent kinase inhibitor 2B
DSRCT: Desmoplastic small round cell tumor
EMA: European Medicines Agency
ES: Ewing sarcoma
EWSR1: Ewing Sarcoma Breakpoint Region 1
FISH: Fluorescence in situ hybridization
FDA: Food and Drug Administration
PDGFRA: Platelet-derived growth factor receptor alpha
HDAC: Histone deacetylase
HDACi: HDAC inhibitor
ΔΨm: Loss of mitochondrial transmembrane potential
MOMP: Mitochondrial outer membrane permeabilization
M-FISH: Multicolor-FISH
mt-p53: Mutant p53
NMPA: National medical products administration
NAD^+^: Nicotinamide adenine dinucleotide
PMDA: Pharmaceuticals and Medical Devices Agency
PNETs: Primitive neuroectodermal tumors
PI: Propidium iodide
qPCR: Quantitative real-time PCR
SirT: Sirtuin
SirTi: SirT inhibitor
TKI: Tyrosine kinase inhibitor
VPA: Valproic acid
VCEP: Variant Curation Expert Panel
VEGF: Vascular endothelial growth factor
wt-p53: Wild-type p53
WT1: Wilms Tumor-1
WT: Wilms tumors
zVAD: Z-VAD-FMK
