# Supplementary material for: Evaluation of novel targeted small-molecule therapeutics in desmoplastic small round cell tumor (DSRCT) cells
Source: J Cancer Res Clin Oncol. 2026 Oct 3;152(10):192. doi: 10.1007/s00432-026-06630-6 (PMC13634027; doi:10.1007/s00432-026-06630-6)

Supplementary figure S1

A

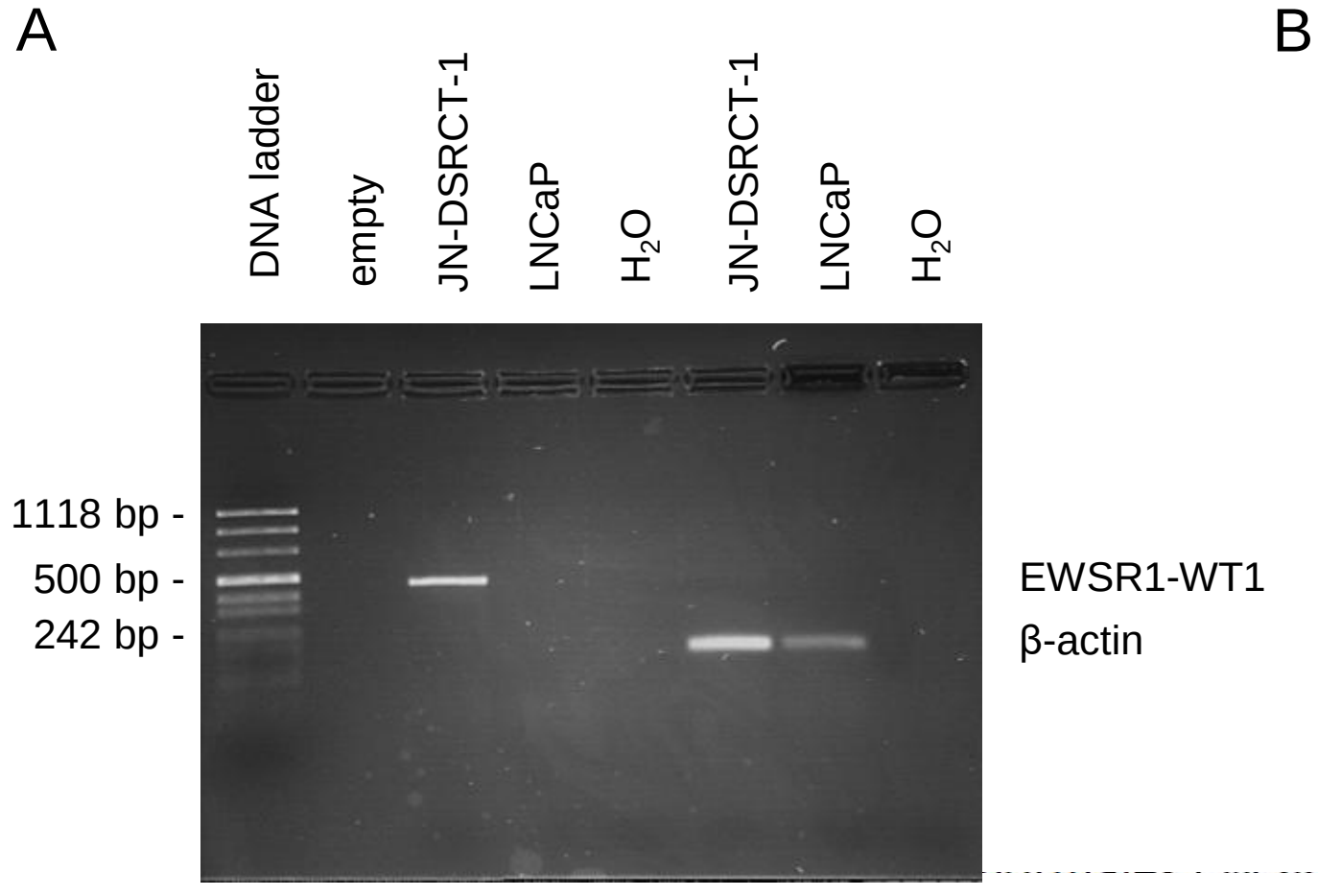


B

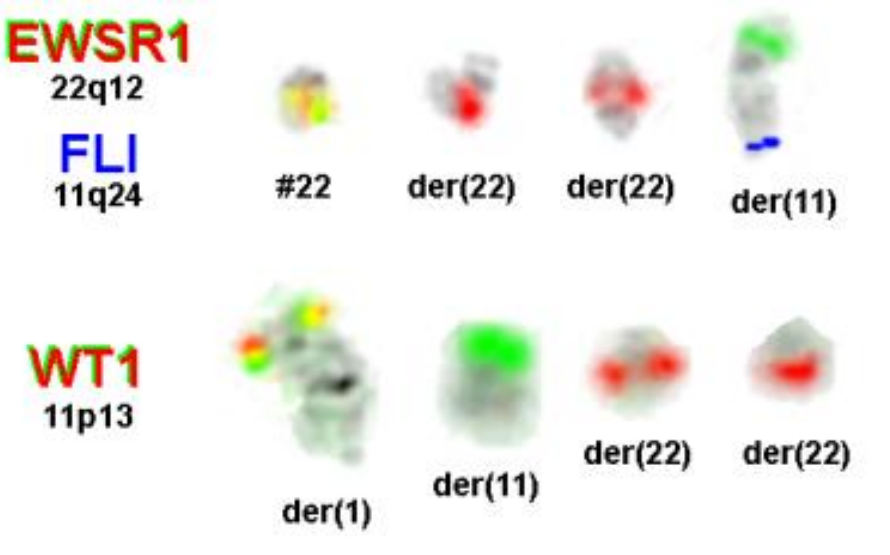


C

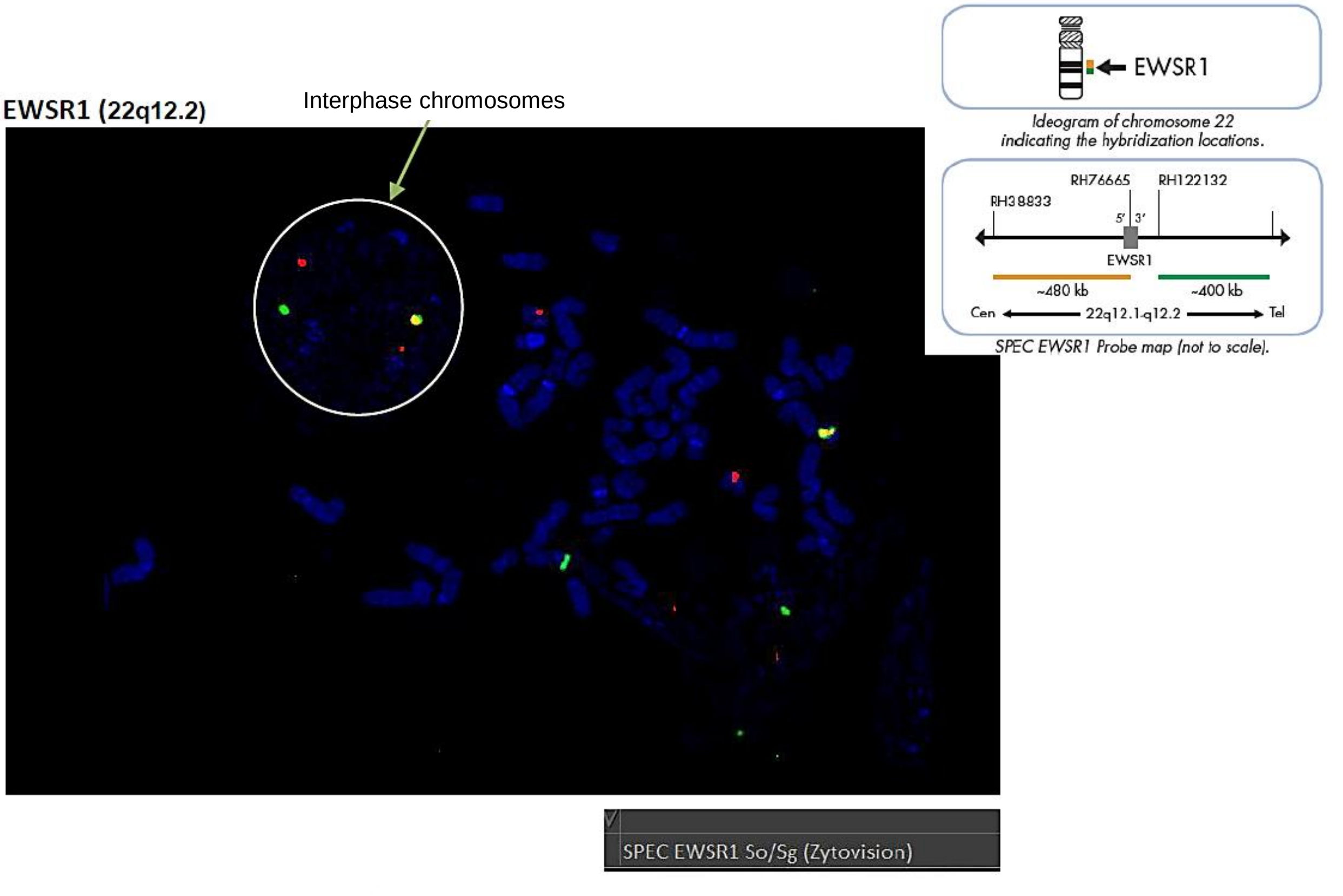


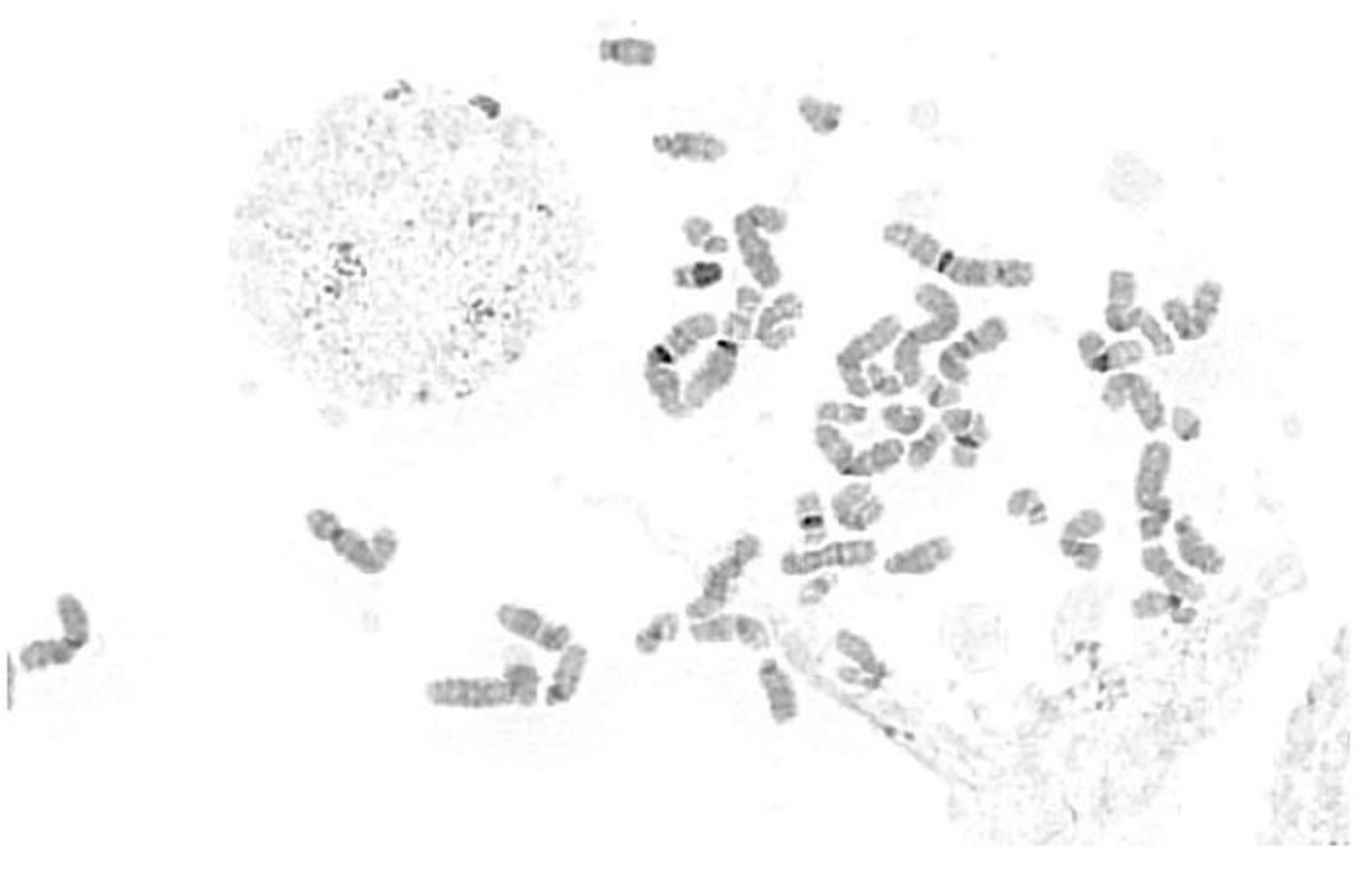

Supplementary figure S2

A

**WT1 (11p13)**

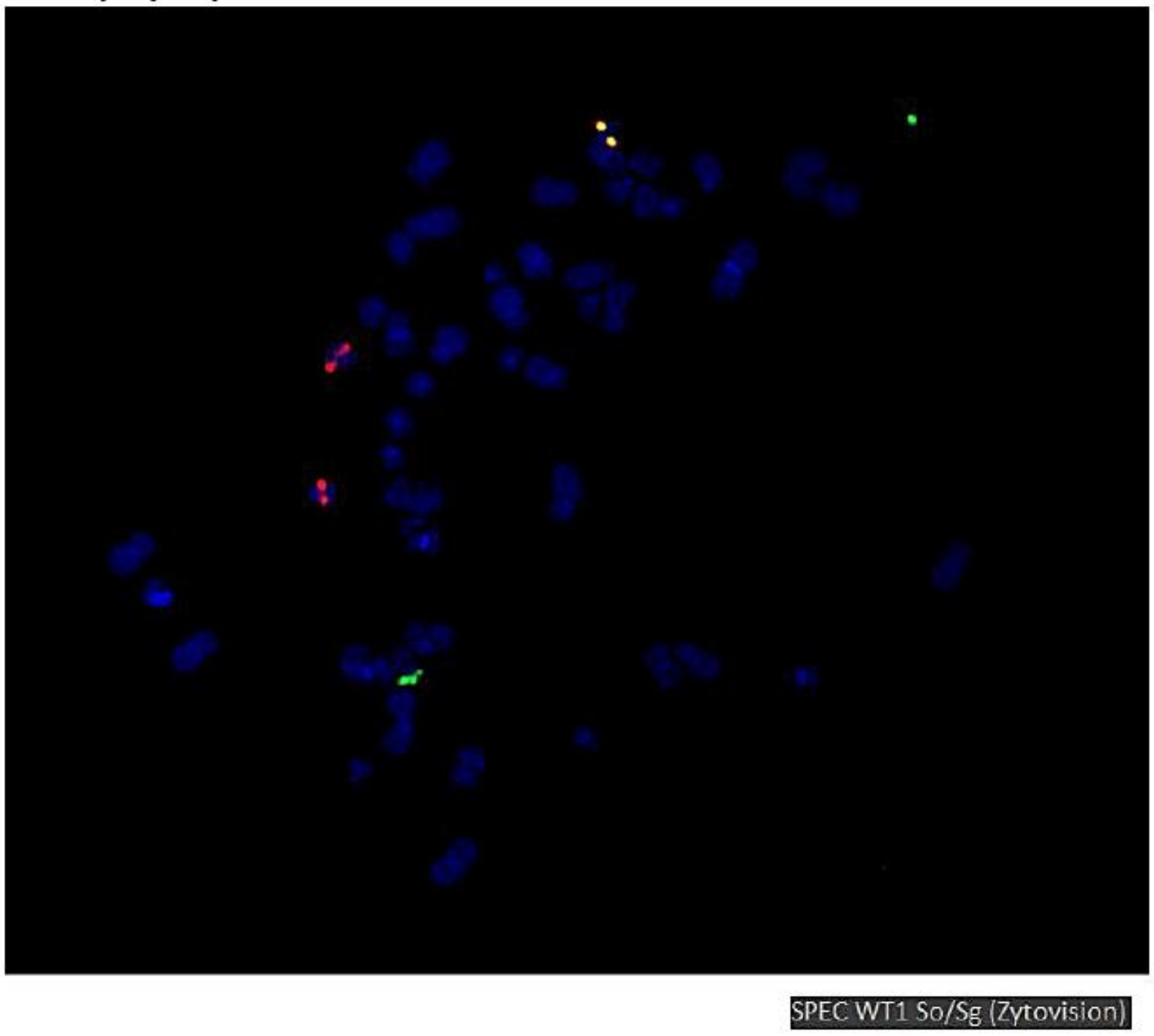


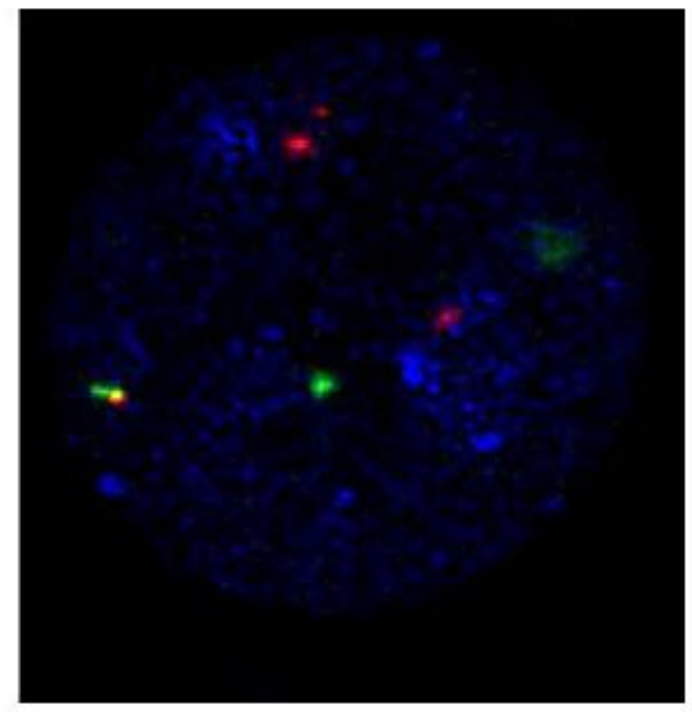

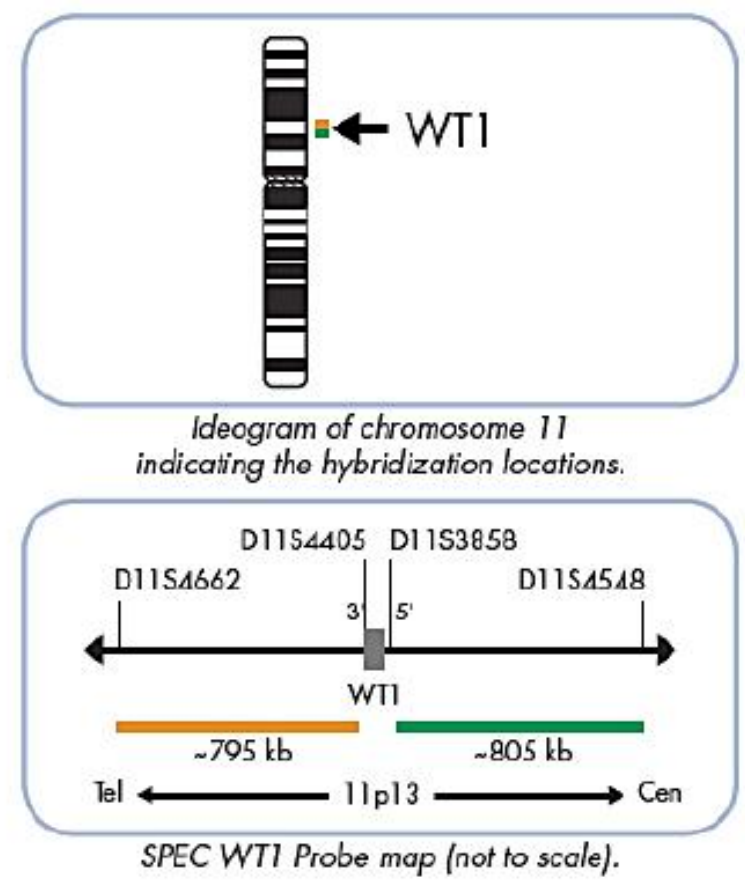


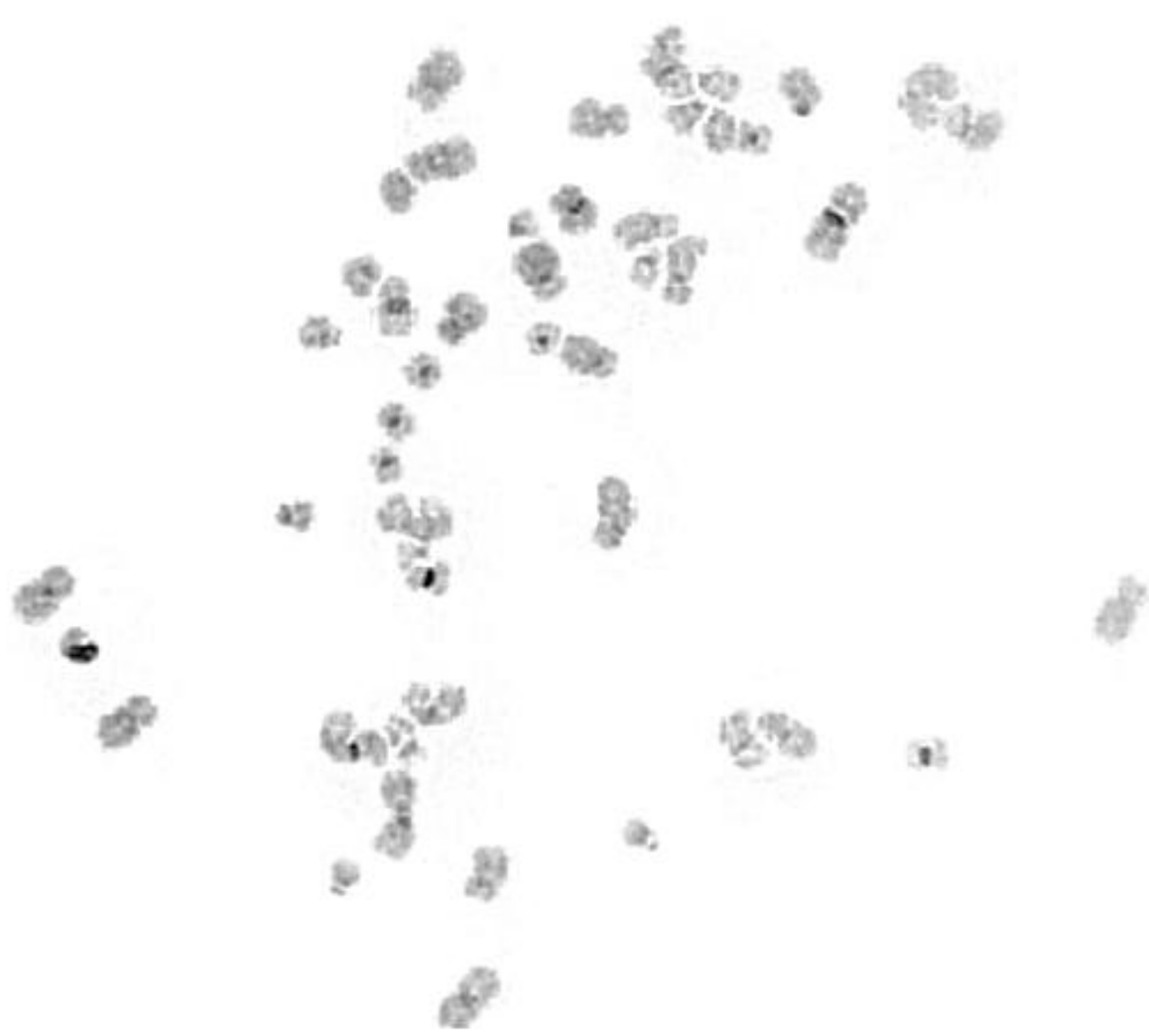

B

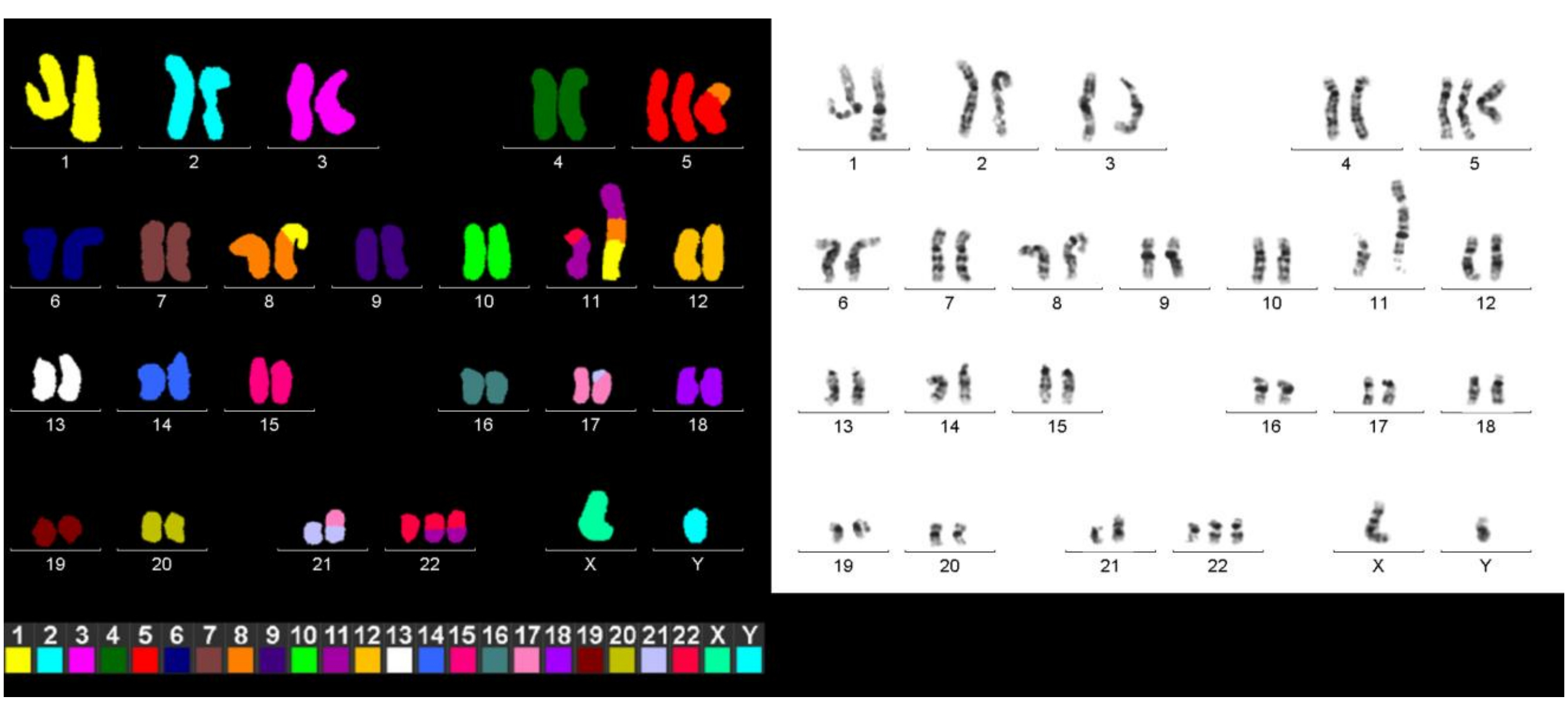

Supplementary figure S3

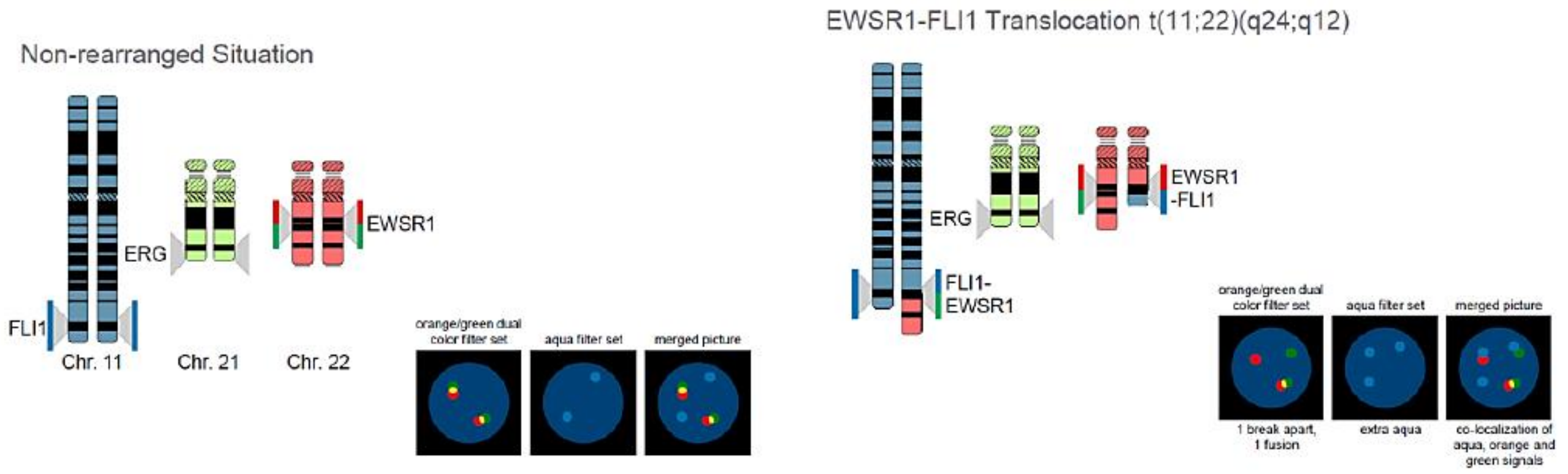


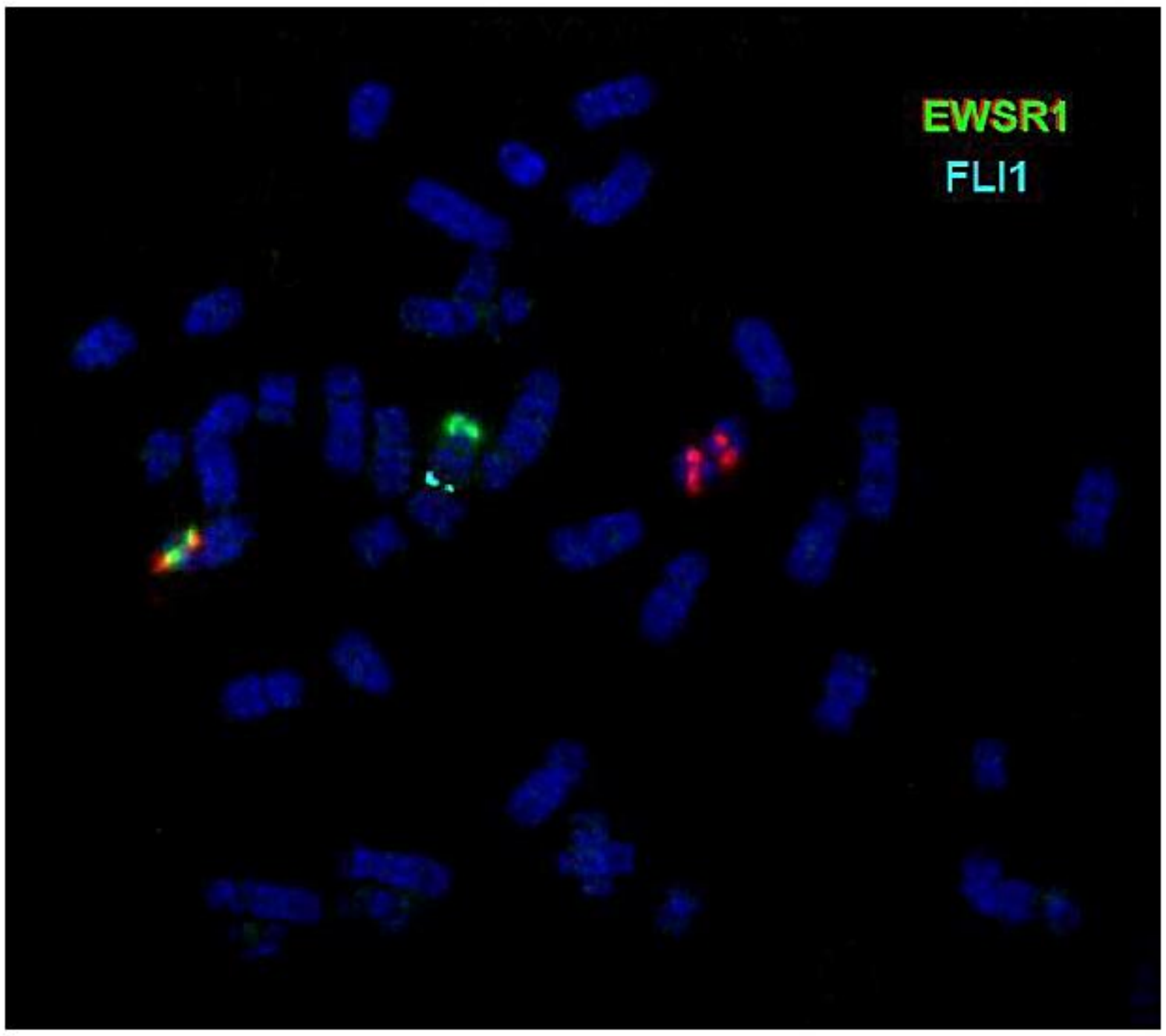

Supplementary figure S4

**Gene:** p53

gene info: ENSG00000141510

transcript info: ENST00000269305

MV: jhen [07/22/2014 09:59:56]

**Result:**

| Index | Location | Position | Type | Nuc Change | AA Change | Web Ref | Mut Ref | Mut Effect |
|---|---|---|---|---|---|---|---|---|
| 1 | E4 | 33 (129) | C | G -> C (het) | L -> F (43) | | | |
| 2 | E4 | 119 (215) | C | C -> G (homo) | P -> R (72) | rs1042522 | c.215C>G | nicht pathogen SNP, G/C 50% |
| | | | | | | | | |

1) E4 33 (129) C G -> C (het) L -> F (43)

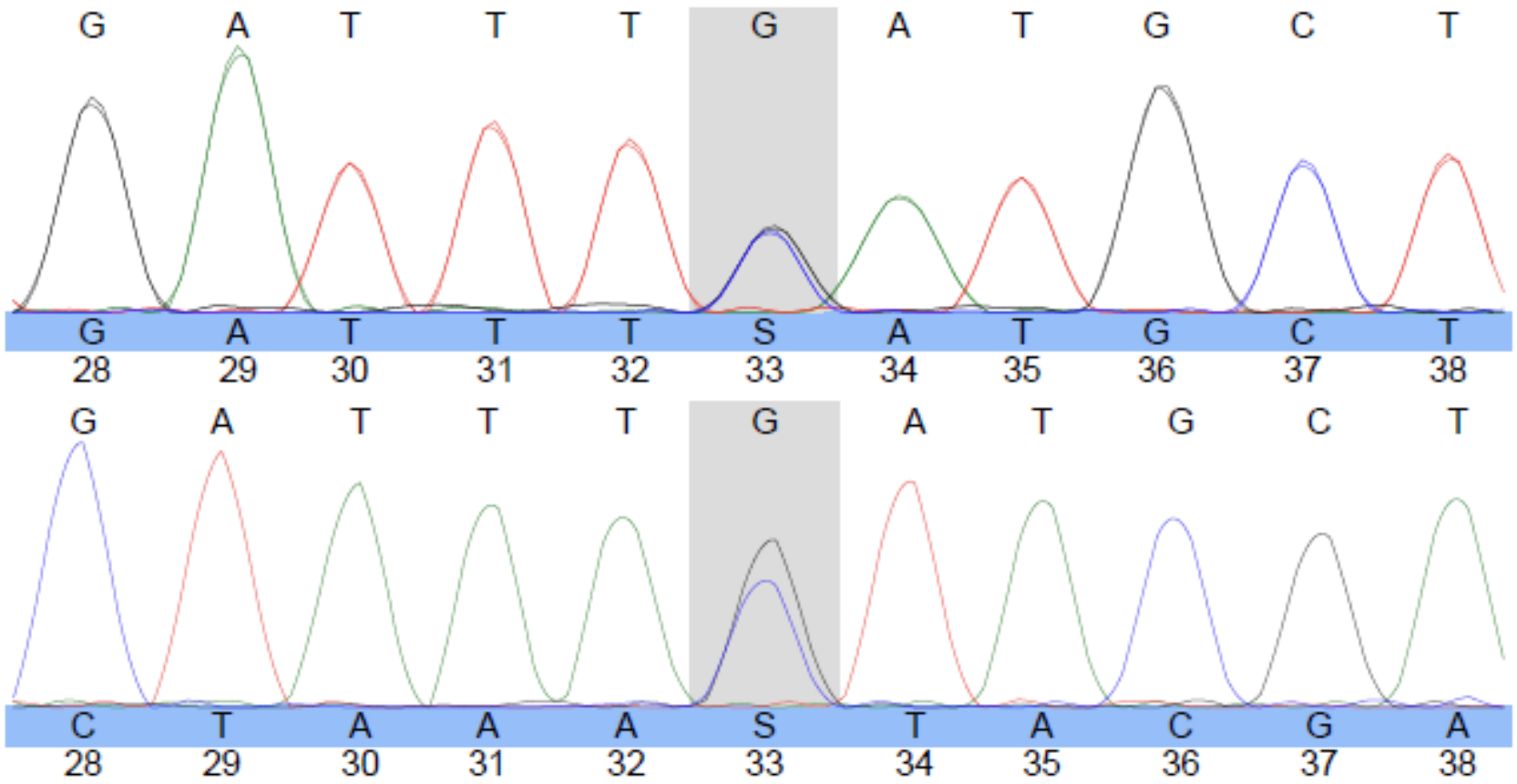

430-14_p53_Ex4_For_F03_06.ab1
Location: E4
Direction: forward
Abs: 95

430-14_p53_Ex4_Rev_C04_03.ab1
Location: E4
Direction: reverse
Abs: 127 / 261

2) E4 119 (215) C C -> G (homo) P -> R (72) rs1042522

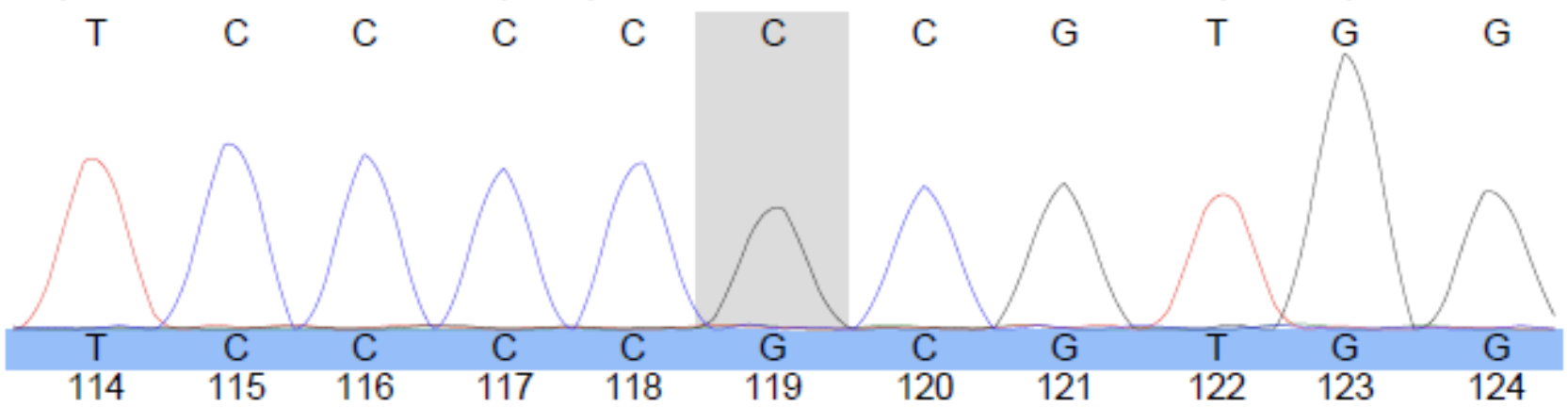

430-14_p53_Ex4_For_F03_06.ab1
Location: E4
Direction: forward
Abs: 181

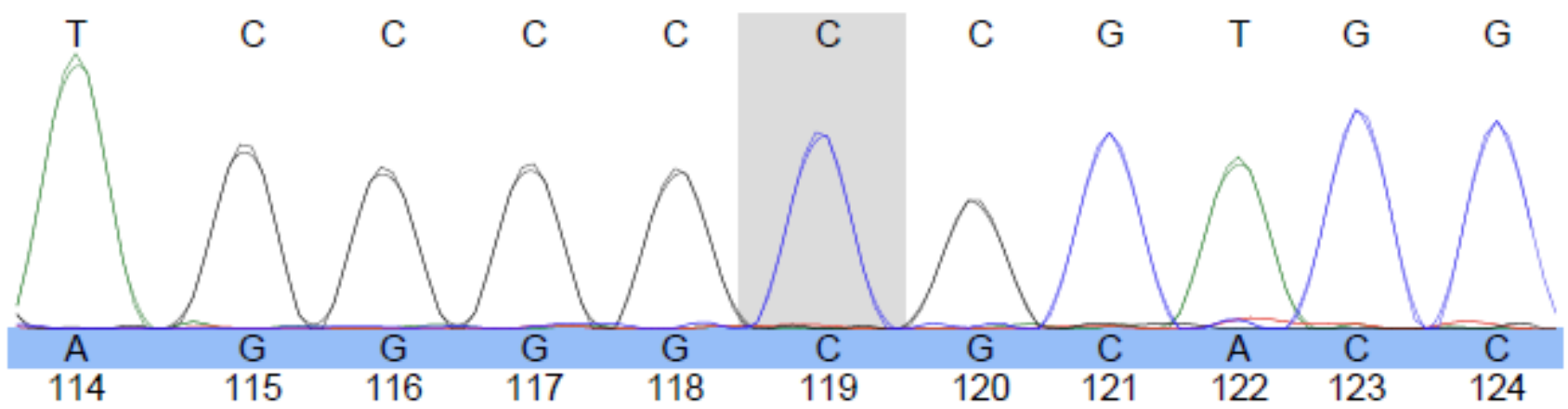

430-14_p53_Ex4_Rev_C04_03.ab1
Location: E4
Direction: reverse
Abs: 213 / 175

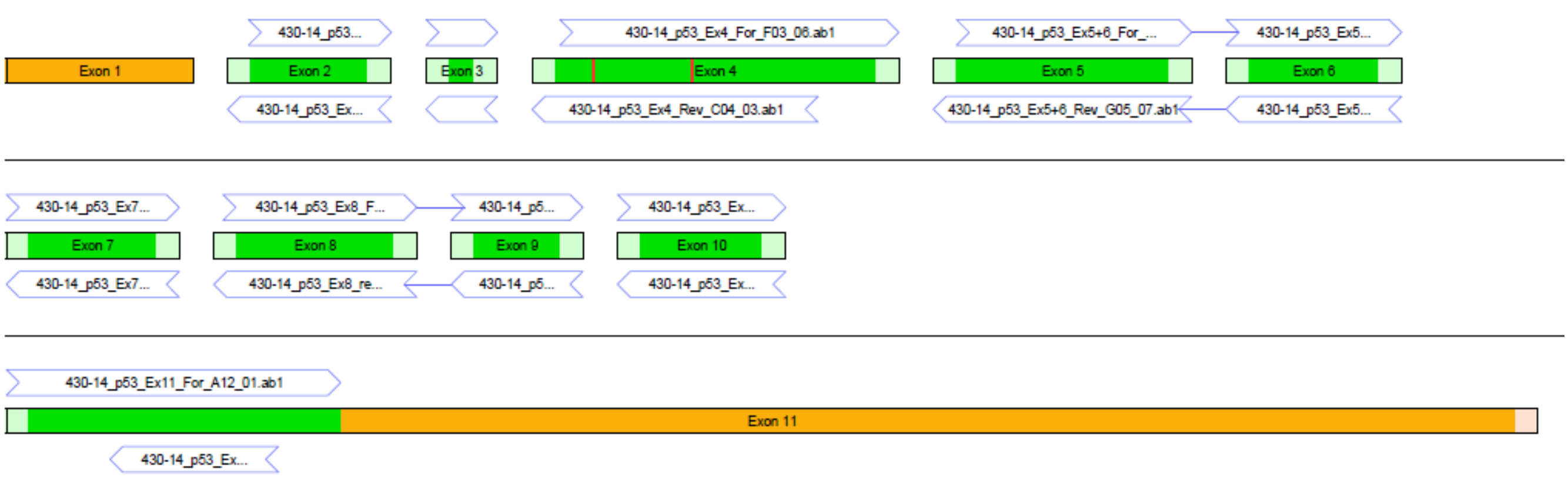

Supplementary figure S5

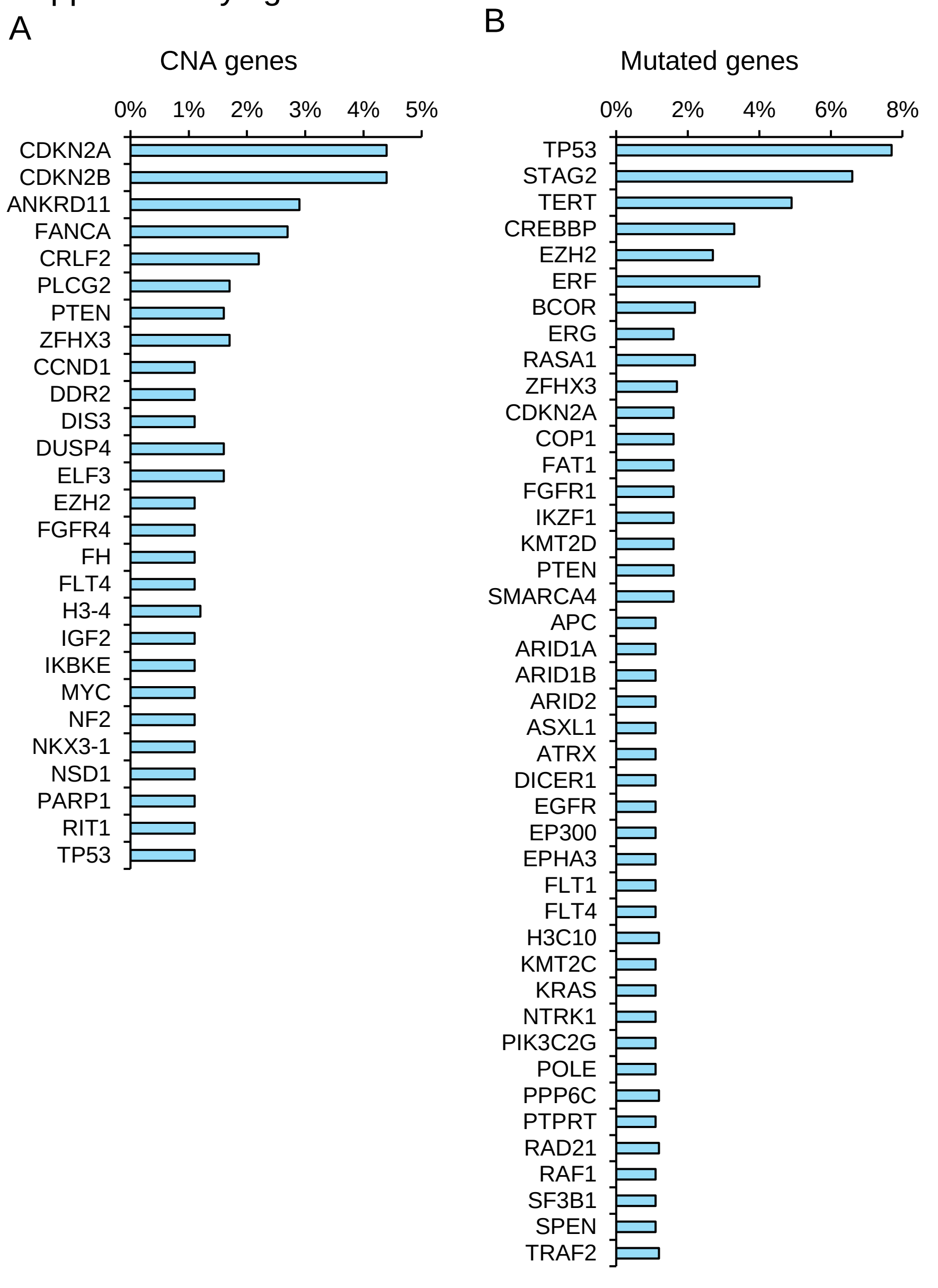

Supplementary figure S6

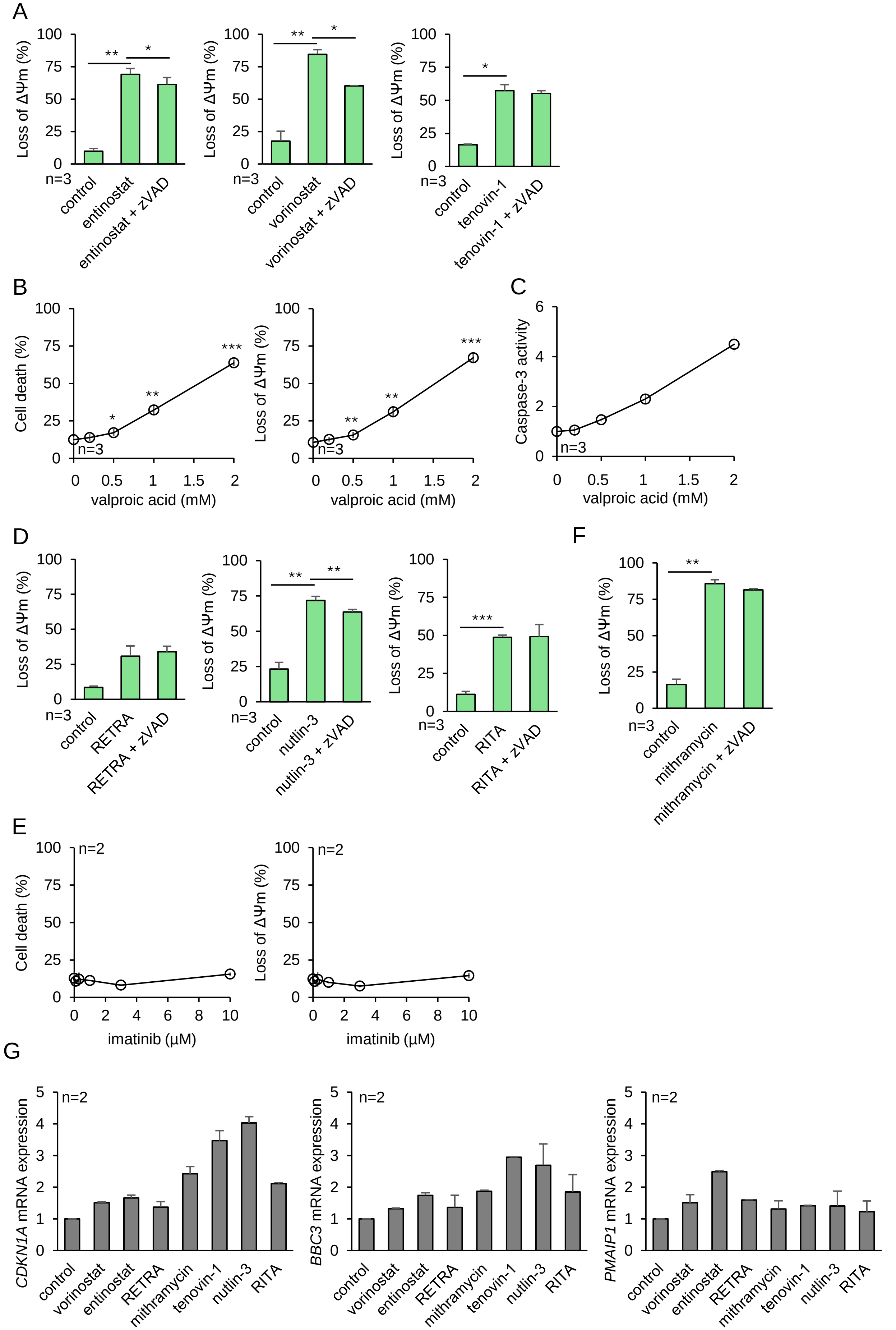

Supplement: Supplementary file 1 [file 432_2026_6630_MOESM1_ESM.pdf]
